# Magnetic Properties of Metal–Organic Coordination Networks Based on 3d Transition Metal Atoms

**DOI:** 10.3390/molecules23040964

**Published:** 2018-04-20

**Authors:** María Blanco-Rey, Ane Sarasola, Corneliu Nistor, Luca Persichetti, Christian Stamm, Cinthia Piamonteze, Pietro Gambardella, Sebastian Stepanow, Mikhail M. Otrokov, Vitaly N. Golovach, Andres Arnau

**Affiliations:** 1 Departamento de Física de Materiales UPV/EHU, 20018 Donostia-San Sebastián, Spain; maria.blanco@ehu.es (M.B.-R.); vitaly.golovach@ehu.es (V.N.G.); 2 Donostia International Physics Center (DIPC), 20018 Donostia-San Sebastián, Spain; ane.sarasola@ehu.es (A.S.); mikhail.otrokov@gmail.com (M.M.O.); 3 Departamento Física Aplicada I, Universidad del País Vasco, 20018 Donostia-San Sebastián, Spain; 4 Department of Materials, ETH Zürich, Hönggerbergring 64, 8093 Zürich, Switzerland; corneliu.nistor@mat.ethz.ch (C.N.); luca.persichetti@mat.ethz.ch (L.P.); christian.stamm@mat.ethz.ch (C.S.); pietro.gambardella@mat.ethz.ch (P.G.); sebastian.stepanow@mat.ethz.ch (S.S.); 5 Swiss Light Source, Paul Scherrer Institute, 5232 Villigen PSI, Switzerland; cinthia.piamonteze@psi.ch; 6 Centro de Física de Materiales (CFM-MPC), Centro Mixto CSIC-UPV/EHU, 20018 Donostia-San Sebastián, Basque Country, Spain; 7 Tomsk State University, Tomsk 634050, Russia; 8 Saint Petersburg State University, Saint Petersburg 198504, Russia; 9 IKERBASQUE, Basque Foundation for Science, 48013 Bilbao, Basque Country, Spain

**Keywords:** magnetism, metal–organic network, X-ray magnetic circular dichroism (XMCD), density functional theory

## Abstract

The magnetic anisotropy and exchange coupling between spins localized at the positions of 3d transition metal atoms forming two-dimensional metal–organic coordination networks (MOCNs) grown on a Au(111) metal surface are studied. In particular, we consider MOCNs made of Ni or Mn metal centers linked by 7,7,8,8-tetracyanoquinodimethane (TCNQ) organic ligands, which form rectangular networks with 1:1 stoichiometry. Based on the analysis of X-ray magnetic circular dichroism (XMCD) data taken at T = 2.5 K, we find that Ni atoms in the Ni–TCNQ MOCNs are coupled ferromagnetically and do not show any significant magnetic anisotropy, while Mn atoms in the Mn–TCNQ MOCNs are coupled antiferromagnetically and do show a weak magnetic anisotropy with in-plane magnetization. We explain these observations using both a model Hamiltonian based on mean-field Weiss theory and density functional theory calculations that include spin–orbit coupling. Our main conclusion is that the antiferromagnetic coupling between Mn spins and the in-plane magnetization of the Mn spins can be explained by neglecting effects due to the presence of the Au(111) surface, while for Ni–TCNQ the metal surface plays a role in determining the absence of magnetic anisotropy in the system.

## 1. Introduction

There exists an exciting type of two-dimensional system that can be grown on surfaces by self-assembly techniques. This is of interest both from a fundamental point of view and because of the potential applications in the fabrication of electronic and spintronic devices. These systems are called metal–organic coordination networks (MOCNs) and consist of metal centers linked by organic ligands that permit, in principle, the design of overlayers with specific electronic and magnetic properties [1]. The synthesis and growth of a given MOCN with a given composition, essentially defined by its stoichiometry and coordination, depends on the relative strength of the interactions between the constituents (organic ligands and metal centers) and their interaction with the underlying surface [2,3,4,5,6,7,8,9,10,11]. Indeed, the chemical state of the organic ligands and metal centers can be modified due to vertical electronic charge transfer from the surface [12]. Additionally, lateral charge transfer between the MOCN constituents is crucial for bonding and equally important for the electronic and chemical properties of the overlayers. Particularly interesting is the role of the metal centers in the formation of the two-dimensional networks by favoring a given coordination and stoichiometry, determining the charge and magnetic moment of the metal center, and, occasionally, also of the organic ligand that can acquire spin polarization. An important point is that this spin-polarized hybrid state could be used to control the electronic and magnetic properties of the interface.

The case of 3d transition metal atoms as metal centers and molecules with large electronegativity, like 7,7,8,8-tetracyanoquinodimethane (TCNQ) or 2,3,5,6-tetrafluoro-7,7,8,8-tetracyanoquinodimethane (F4TCNQ), on metal surfaces is of special interest because they form well-ordered MOCNs with few defects and different stoichiometry [4,7,13]; the latter characteristic depends both on the underlying surface and preparation conditions. The experimental techniques typically used to characterize the geometric structure and chemical composition of MOCNs on surfaces are scanning tunneling microscopy (STM), low-energy electron diffraction (LEED), and X-ray photoemission spectroscopy (XPS), while for the electronic and magnetic properties the standard techniques are angle-resolved photoemission spectroscopy (ARPES) , scanning tunneling spectroscopy (STS), and X-ray magnetic circular dichroism (XMCD).

The theoretical description of this type of system has been shown to be quite reasonable using standard electronic structure methods, like density functional theory (DFT) [4,11,13,14], although one has to be aware of the limitations of each method when aiming at achieving quantitative agreement with the experimental data. However, the explanation of the observations at a qualitative level and its understanding with the help of a model Hamiltonian is the recipe that we follow in this work. In any case, it is worth mentioning that an essential problem, which is hard to overcome, concerns the accuracy of the calculations when dealing with very low energy scales, as is the case of the determination of exchange couplings or magnetic anisotropies in the sub-meV energy range. Apart from this limitation imposed by the methodology itself, it is also important to stress that exchange coupling and magnetic anisotropy energy are extremely sensitive to both slight geometrical distortions and band filling, i.e., electronic charge transfer. Therefore, it is important to balance the different effects that each and every approximation can have in the final results when trying to explain an observation that can be deduced, e.g., from XMCD data, regarding the strength and type (ferro or antiferromagnetic) of exchange coupling or the kind of magnetic anisotropy (easy axis or easy plane).

The exchange coupling between magnetic centers in two-dimensional MOCNs is affected to a major or lesser extent by the underlying substrate. The presence of the surface represents a difficulty for describing the full system (MOCN/surface) because the overlayer structure is not necessarily commensurable with the crystal surface or because the size of the commensurable supercell is too large. In the case of weak coupling between the overlayer and the surface, as is the case of Au(111) surfaces, the essential features can be described by neglecting the role of surface electrons in the first approximation. As a rule of thumb, when the lateral bonds between the metal centers and the organic ligands are much stronger than the metal–surface or ligand–surface bonds, this approximation is expected to be a reasonable way to describe the magnetic coupling between metal atoms in the MOCN. However, in case of lateral (metal–molecule or intermolecular) and vertical (MOCN–surface) couplings of comparable strengths, an explicit inclusion of the surface is required to describe the system, which could happen either due to strong coupling with the surface or weak lateral coupling. Next, one should consider the role of charge transfer between surface and overlayer, even in the case of weak coupling, as it can be important in determining magnetic moments, magnetic coupling, or even magnetic anisotropy. Indeed, when intermolecular coupling is weak, the role of surface electrons can be relatively more important in determining the magnetic coupling between spins of the metal centers via Ruderman–Kittel–Kasuya–Yosida (RKKY) interaction [15], which may appear not only on metal surfaces but also on the surface of topological insulators [16,17]. Very recently, it has been proposed that the RKKY interaction is responsible for the long-range ferrimagnetic order in a two-dimensional Kondo lattice with underscreened spins by the conduction electrons in a FeFPc–MnPc mixture on the Au(111) surface [18].

In this work, we consider the case of MOCNs that consist of Mn or Ni magnetic atoms and TCNQ or F4TCNQ molecules grown on Au(111) surfaces, which show 1:1 stoichiometry with each metal center (Mn or Ni) coordinated with four organic ligands. Our preliminary study of the systems Ni–TCNQ and Mn–TCNQ on Au(111) [11] was focused on the type of exchange coupling between Ni and Mn centers, in fact showing important differences between Ni and Mn networks in XMCD data taken at T=8 K, the most significant being that Mn (Ni) metal atoms are antiferro (ferro) magnetically coupled. Now, for these systems, our new XMCD data taken at a lower temperature (T=2.5 K) reveal additional information about the magnetic anisotropy in the systems and, therefore, we have performed new first principle calculations including spin–orbit coupling (SOC) to explain the results of the observations. We have also considered the role of the Au(111) metal surface, which can introduce geometrical distortions in the networks and electronic charge exchange with its constituents. Additionally, we have developed a more refined model that may account for magnetic frustration in the systems as well, by including exchange coupling up to next nearest neighbors. The results of our calculations for the free-standing neutral Mn–TCNQ overlayers are consistent with both the antiferromagnetic coupling between Mn centers and the weak magnetic anisotropy with in-plane magnetization, while for Ni–TCNQ overlayers we need to call for effects due to the presence of the underlying metal surface, like charge transfer and changes in coordination, to explain the absence of anisotropy in the system. Model calculations based on mean-field Weiss theory permit us to extract exchange coupling constants from the fits to XMCD curves, as well as to obtain additional information about the magnetic anisotropy and the different magnetic configurations that may appear in the networks. Here, we do not aim at achieving quantitative agreement between the fitting parameters (exchange coupling constants) used for the XMCD curves and those extracted from DFT total energy calculations but we do give an explanation for the differences observed, these being large for Ni–TCNQ. Finally, it is worth mentioning that, although the organic ligands TCNQ and F4TCNQ have a different electronegativity (higher in F4TCNQ than in TCNQ), based on the acquired XMCD data, there are no substantial differences in the magnetic properties of the corresponding Ni and Mn networks. Therefore, in the core of the paper we present the results for TCNQ networks and leave the F4TCNQ results for Section II of the Supplementary Material.

The paper is organized as follows. After describing the XMCD experiments and the technical details of the calculations in Section 2, we present our XMCD data for Mn–TCNQ and Ni–TCNQ on Au(111) in Section 3.1, together with fitting curves from model calculations that permit us to explain the observations and extract information about the type of magnetic coupling and magnetic anisotropy (Section 3.2). Next, in Section 3.3 we present the results of our spin-polarized DFT+U electronic structure calculations for Mn–TCNQ and Ni–TCNQ free-standing overlayers that confirm the observed behavior in the type of magnetic coupling between spins at the 3d metal centers. Then, in Section 3.4, we present the magneto-crystalline anisotropy analysis of the two considered systems under study based on calculations that include spin–orbit coupling. Finally, in Section 4, we present a discussion of our findings and establish the main conclusions that aim at explaining the XMCD observations and suggest that for Ni–TCNQ networks the Au(111) metal surface plays a role in determining the magnetic properties of the MOCN, while this is not the case for Mn–TCNQ.

## 2. Materials and Methods

### 2.1. X-ray Magnetic Circular Dichroism Experiments

X-Ray absorption spectroscopy (XAS) experiments were carried out at the X-Treme beamline of the Swiss Light Source (Villigen, Switzerland) [19]. The samples were prepared in ultra-high vacuum chambers with a base pressure in the range of low 10${}^{-10}$ mbar. The pressure in the magnet-cryo-chamber was always better than 10${}^{-11}$ mbar. The Au(111) surface was cleaned by repeated cycles of Ar${}^{+}$ sputtering and subsequent annealing to 800 K. The molecules 7,7,8,8-tetracyanoquinodimethane (TCNQ, 98% purity, Aldrich, Saint Louis, MO, USA) and 2,3,5,6-tetrafluoro-7,7,8,8-tetracyanoquinodimethane (F4TCNQ, 97% purity, Aldrich) were thoroughly degassed before evaporation. The organic adlayers were grown by organic molecular beam epitaxy (OMBE) using a resistively heated quartz crucible at a sublimation temperature of 408 K onto the clean Au(111) surface that was kept at room temperature. The coverage of molecules was controlled to be below one monolayer. Ni or Mn was subsequently deposited using an electron beam heating evaporator at a flux of about 0.01 ML/min on top of the molecular adlayers that were heated to 350–400 K to promote the network formation. The sample was checked in situ by STM at the beamline and subsequently transferred to magnet chamber without breaking the vacuum. A representative STM image, which shows the typical Mn-TCNQ network domains on Au(111), can be found in the Supplementary Material.

**Figure 1:**
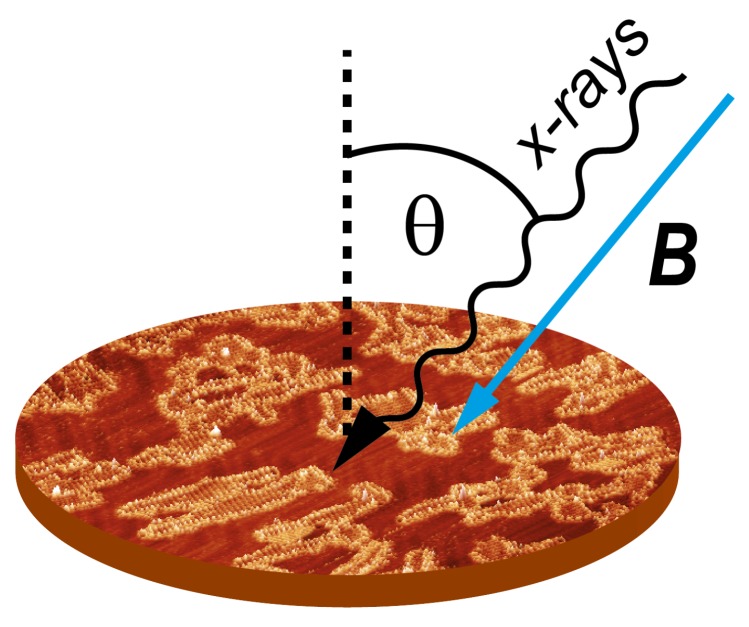
Schematic view of the data acquisition geometry in the X-ray absorption spectroscopy (XAS) experiments. The external magnetic field B is kept parallel to the incident beam and the surface is rotated at a polar angle θ with respect to the surface normal.

The polarization-dependent XAS experiments were performed in total electron yield detection. Magnetic fields were applied collinear with the photon beam at sample temperatures between 2.5 and 300 K. The data were acquired by varying the photon energy at the L${}_{2,3}$ edges of Ni and Mn, as well as the K edges of O and N using circular and linear polarized light. The absorption spectra were normalized with respect to the total flux of the incoming X-rays and were further treated to be normalized to the absorption pre-edge due to total electron yield variations. The background obtained from clean or molecule-covered Au(111) was subtracted to allow comparison of the spectral features. The XMCD is obtained from the difference of the left and right circular polarized XAS spectra, whereas the XAS is obtained from the average of the two circular polarizations. The sample was rotated between normal X-ray incidence with respect to the sample surface at $\theta =0^{\circ }$ and grazing incidence with $\theta =60^{\circ }$ (see Figure 1). All shown spectra were acquired at T=2.5 K at external magnetic fields up to $\mu_{0}H=6.8$ T. The magnetization curves were recorded by acquiring the maximum of the XMCD signal at the L${}_{3}$ edge as a function of the external magnetic field, normalized by the corresponding pre-edge of the XAS signal. To facilitate the extraction of the easy and hard magnetization axes, the magnetization curves at different angles of the magnetic field were normalized to the same value at the highest magnetic field point.

### 2.2. Density Functional Theory Calculations

DFT calculations were carried out using the Vienna Ab Initio Simulation Package (VASP) [20,21,22]. For the description of electron–ion interactions the projector augmented wave (PAW) method was employed, whereas the Perdew, Burke, and Ernzerhof (PBE) functional was used to describe exchange and correlation within the generalized gradient approximation (GGA) [23]. A Hubbard-like Coulomb repulsion correction term (U = 4 eV) was added to describe the 3d metal electron states, based on Dudarev’s approach [24] , as implemented in VASP. A previous study [11] has already corroborated that the results concerning magnetic moments and 3d level occupations do not change appreciably in the 3–5 eV range of the U parameter.

For the geometrical optimization of the free-standing Mn–(F4)TCNQ and Ni–(F4)TCNQ systems, periodic supercell boundary conditions were imposed. The optimal cell dimensions and atomic positions were obtained by an energy minimization procedure with a convergence criterion of $10^{-6}$ eV for the energy and 0.02 eV/Å for the forces to assure that we reach sufficient accuracy in numerical values of the calculated magnitudes. The Kohn–Sham wave functions were expanded in a plane wave basis set with a kinetic energy cutoff of 400 eV for all the systems considered. Monkhorst–Pack k-point sampling equivalent to 8×12 in the 1×1 surface unit cell [25] and Methfessel–Paxton integration with smearing width 0.1 eV [26] were used. Symmetry considerations were switched off from the calculations and a preconverged charge density with a fixed value of the total spin for the unit cell was used to relax all the networks. For the obtained relaxed 1×1 geometries, where the layer is constrained to be flat, we evaluated the magnetic anisotropy energies with adjusted parameters. Total energies were converged with a tolerance of $10^{-7}$ eV. A 12×18 k-point sampling and the corrected tetrahedron method of integration [27] were used instead of smearing methods.

**Figure 2:**
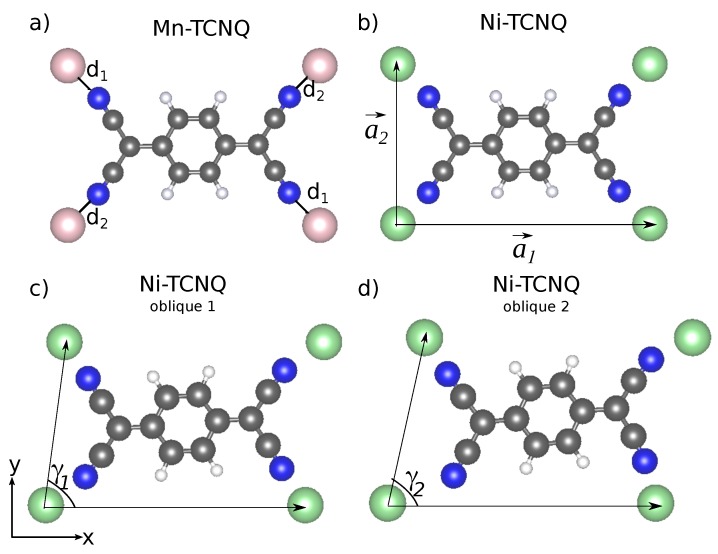
Visualization of the Mn–TCNQ (**a**) and Ni–TCNQ (**b**) rectangular cells. Blue, gray, and white circles correspond to N, C, and H atoms respectively, while bright violet and bright green circles correspond to Mn and Ni atoms. The fluorinated (F4)TCNQ molecules differ from regular TCNQ only in having F atoms instead of H, the corresponding C–F bond lengths being somewhat longer than those of C–H. Panels (**c**,**d**) show the distorted cell models used for Ni–TCNQ. Geometry details are found in Table 1. TCNQ, 7,7,8,8-tetracyanoquinodimethane.

Figure 2 shows a top view visualization of the rectangular and oblique cells considered. The optimized geometrical parameters are included in Table 1, where $\vec{a_{1}}$ and $\vec{a_{2}}$ denote the lattice vectors, *a${}_{1}$* and *a${}_{2}$* their moduli, while d${}_{1}$ and d${}_{2}$ denote the values of the Mn–N or Ni–N bond lengths indicated in Figure 2.

## 3. Results

### 3.1. X-ray Magnetic Circular Dichroism Data

The XMCD intensity variation as a function of the applied magnetic field (*B*) defines a curve that is proportional to the system magnetization. Therefore, when the value of the spin magnetic moments at the metal centers (*S*) , the temperature (*T*), and the Landé *g*-factor are known, one can use simple models to simulate the magnetization response. A good reference to be considered is the case of paramagnetic behavior (spins responding individually to the applied magnetic field) that can be represented by a Brillouin function. Whenever a preference for ferromagnetic (FM) or antiferromagnetic (AFM) coupling between spins appears, the corresponding magnetization curves will show higher or lower curvature, respectively, than the corresponding Brillouin function for the same *S*, *T*, and *g*-factor values. In this way, in principle, one can decide about the type of magnetic coupling between localized spins at the metal centers, as long as the value of the spin (*S*) is known. Note that, in the presence of strong magnetic anisotropies and high orbital angular moments, the analysis becomes more involved [28]. However, here we can follow this simplified scheme, as shown below. According to our DFT calculations, described in Section 3.3, Mn atoms in Mn–TCNQ have a localized spin magnetic moment close to S=5/2, although somewhat lower, while Ni atoms in Ni–TCNQ have a much lower spin close to S=1/2, although somewhat higher. Therefore, we use the values S=5/2 and S=1/2 for Mn and Ni, respectively, to perform our XMCD analysis that includes fitting curves to XMCD data based on Weiss mean-field theory described in the next section, where *J* and *D* are defined, and also a comparison with the corresponding Brillouin functions.

**Figure 3:**
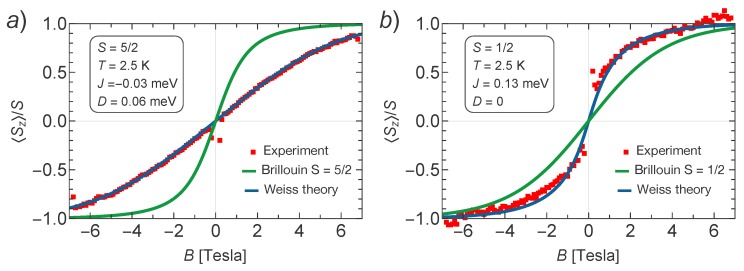
The best fit with the Weiss mean-field theory to the experimental data for (**a**) Mn–TCNQ and (**b**) Ni–TCNQ at normal beam incidence (θ=0°) and the temperature T=2.5K. The experimental data are shown in red squares, whereas the solution of the mean-field self-consistency equations is shown as the blue solid curve. For comparison, we also plot the Brillouin function for S=5/2 in (**a**) and S=1/2 in (**b**), showing that the shape of the measured magnetization versus *B* deviates substantially from the Brillouin function at this temperature.

**Figure 4:**
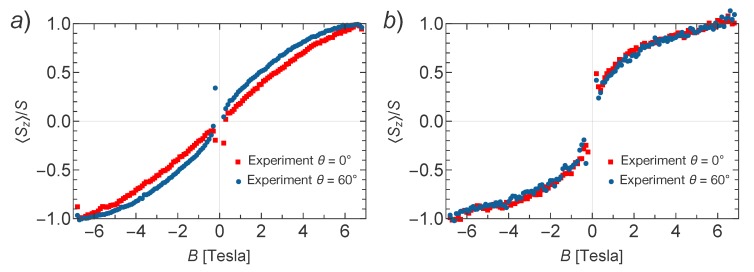
Comparison of the rescaled X-ray magnetic circular dichroism (XMCD) signal measured for (**a**) Mn–TCNQ and (**b**) Ni–TCNQ at normal (θ=0°) and grazing (θ=60°) beam incidences. The data in (**a**) show a sizable θ-dependence, which we attribute to the single-ion anisotropy for Mn–TCNQ. In contrast, the data in (**b**) show no θ-dependence, meaning that there exists no sizable magnetic anisotropy.

The results are shown in Figure 3a,b for Mn–TCNQ and Ni–TCNQ, respectively. It is evident that in Mn–TCNQ the coupling between Mn spins is AFM, while in Ni–TCNQ it is FM. Additionally, the fitted values of the exchange coupling constants reveal a weaker coupling between Mn spins (J=−0.03 meV) as compared to the coupling between Ni spins (J=0.13 meV), while the single ion anisotropy parameter D=0.06 meV corresponds to a weak anisotropy with in-plane magnetization for Mn–TNCQ and D=0 to the absence of anisotropy for Ni–TCNQ. In order to learn more about the magnetic anisotropy of these systems, in Figure 4 we plot a comparison of XMCD data obtained for perpendicular and grazing incidence for Mn–TCNQ and Ni–TCNQ, the former showing a mild angular dependence with stronger intensity for grazing incidence, i.e., a fingerprint of magnetic anisotropy in the system with in-plane magnetization. Incidentally, this weak anisotropy is only observed at low temperatures. However, in the Ni–TCNQ XMCD data there is no significant angular dependence, which means a negligible magnetic anisotropy. A value of the Ni atom spin S=1/2 corresponds to the absence of single ion anisotropy [29].

### 3.2. Model for Mn–TCNQ and Ni–TCNQ

**Figure 5:**
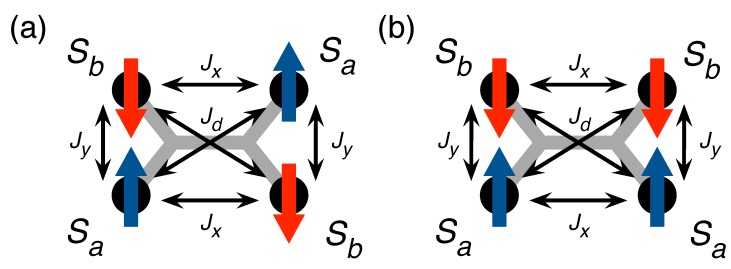
Sketch of the Mn–TCNQ lattice showing the relevant magnetic couplings between the Mn atoms. The four-leg TCNQ molecules mediate by superexchange an antiferromagnetic interaction between the nearest neighbors on the lattice (couplings $J_{x}$ and $J_{y}$) as well as between the next-to-nearest neighbors (coupling $J_{d}$). For a sufficiently small-magnitude $J_{d}$, the tendency is to order the spins in the checkerboard pattern (**a**). With increasing the magnitude of $J_{d}$, a crossover to ordering spins in rows or columns takes place (**b**).

In Mn–TCNQ, the coupling between local moments is antiferromagnetic and occurs by means of the Anderson superexchange mechanism [15,30]. In perturbation theory, the superexchange interaction was found to be dominated by a virtual process in which two electrons hop from the lowest unoccupied molecular orbital (LUMO) of the TCNQ molecule, which is doubly occupied in this MOCN, onto two adjacent Mn atoms [11]. Inclusion of additional molecular orbitals, such as the highest occupied molecular orbital (HOMO), leads to a generic superexchange interaction with coupling constants $J_{x}$, $J_{y}$, and $J_{d}$, as shown in Figure 5. The model Hamiltonian describing the magnetic properties of Mn–TCNQ, thus, reads

(1) $$H=-\frac{1}{2}\sum_{ij}J_{ij}S_{i}\cdot S_{j}+D\sum_{i}S_{i,z}^{2}+g\mu_{B}\sum_{i}S_{i}\cdot B,$$

where $S_{i}$ denotes the local moment of the Mn atom (S=5/2) on site *i*, *D* is the single-ion anisotropy energy, *g* is the Landé *g*-factor (g≈2), and B is the magnetic field. The Heisenberg exchange constant $J_{ij}$ is restricted to the nearest ($J_{x}$ and $J_{y}$) and next-to-nearest ($J_{d}$) neighbors on the rectangular lattice. The summation in the Heisenberg interaction term accounts twice for each pair of interacting sites; hence the presence of the factor 1/2 in Equation (1).

A quick insight into the tendency to order the spins in this model is granted by the Fourier transform of the exchange coupling $J_{ij}$,

(2) $$\begin{array}{ccc} J_{q} & = & \sum_{j}J_{ij}e^{-iq\cdot \left(r_{j}-r_{i}\right)}=2J_{x}\cos\left(q_{x}a_{x}\right)\\ & & +2J_{y}\cos\left(q_{y}a_{y}\right)+4J_{d}\cos\left(q_{x}a_{x}\right)\cos\left(q_{y}a_{y}\right), \end{array}$$

where $q=(q_{x},q_{y})$ is the two-dimensional wave vector and $r_{i}$ is the position of the Mn atom on site *i*. For ferromagnetic couplings ($J_{ij}>0$), the maximum of $J_{q}$ occurs at q=0, which indicates that the spin order could be uniform from a mean-field point of view, not addressing the question about its stability against fluctuations in two dimensions. Additional terms, such as the single-ion anisotropy or the Zeeman interaction, may stabilize the uniform spin order.

In contrast, for antiferromagnetic couplings ($J_{ij}<0$), the maximum of $J_{q}$ occurs usually at the edge of the Brillouin zone, indicating that the magnetization is staggered in some way over the unit cell. When only nearest neighbors are coupled ($J_{x}=J_{y}\neq 0$ and $J_{d}=0$), the maxima lie at $q=(\pi /a_{x},\pi /a_{y})$ and its equivalent points, which results in the usual checkerboard-like antiferromagnetic order (see Figure 5a). As the diagonal coupling is turned on (assuming an antiferromagnetic $J_{d}<0$), for a sufficiently large magnitude of $J_{d}$ there is a transition from the checkerboard pattern to a so-called superantiferromagnetic state of antiferromagnetically ordered rows or columns. For $\left|J_{y}\right|>\left|J_{x}\right|$, by requiring $\partial J_{q}/\partial q_{x}\equiv 0$ at $q_{y}=\pi /a_{y}$, we find at $J_{d}=J_{x}/2$ the transition point for antiferromagnetic column formation (see Figure 5b).

The effect of the diagonal coupling $J_{d}$ consists in introducing magnetic frustration [30,31] in the spin lattice. We remark here that the special point $J_{d}=J_{x}/2$ is realized to a good approximation in our Mn–TCNQ lattice, because (1) the LUMO of the TCNQ molecule has a weak overlap with the d${}_{xz}$ and d${}_{yz}$ orbitals of the Mn atom, as will be shown in the next section, thus, dominating the superexchange, and (2) the direct coupling between the LUMOs of neighboring TCNQ molecules is rather weak. The latter makes it possible to consider two independent paths of superexchange for the nearest neighbors, with each path going separately via one of the two TCNQ molecules connecting the two neighboring Mn atoms. For the diagonal coupling, only one path is possible, which leads to a reduction of the diagonal coupling by a factor of 2 as compared to the nearest-neighbor coupling. With approximations (1) and (2), the coupling constants obey $J_{x}=J_{y}=2J_{d}$ (see [11] for further details).

Despite the fact that the Mn–TCNQ lattice may well be in a frustrated magnetic state consisting of a mixture of the two phases in Figure 5, the XMCD data appear to be consistent with a much simpler description of the magnetization as a function of the *B*-field, which is derived from the Weiss mean-field theory, and it faithfully captures weak deviations from the paramagnetic state. The superexchange couplings are rather weak [11], of the order of $10^{-5}\;\mathrm{eV}$, and the Zeeman term soon dominates. Additionally, there exists a fair amount of single-ion anisotropy, described by the $DS_{z}^{2}$ term in Equation (1).

We make the mean-field approximation for the model in Equation (1),

(3) $$\begin{array}{c} H\approx H_{\mathrm{MF}}:=H_{\mathrm{loc}}+\frac{1}{2}\sum_{ij}J_{ij}\langle S_{i}\rangle \cdot \langle S_{j}\rangle ,\\ H_{\mathrm{loc}}=\sum_{i}S_{i}\cdot h_{i}+D\sum_{i}S_{i,z}^{2},\\ h_{i}=g\mu_{B}B-\sum_{j}J_{ij}\langle S_{j}\rangle , \end{array}$$

where $H_{\mathrm{loc}}$ gives the local description of the interacting system in terms of the Weiss fields $h_{i}$. The spin averages $\langle S_{i}\rangle$ can be regarded as variational parameters of the theory. The last term in the first line of Equation (3) compensates for the double counting of interaction energy occurring in the local Hamiltonian $H_{\mathrm{loc}}$ and plays an important role when calculating the free energy of the interacting system. The minimization of the free energy allows us to determine the values of the order parameters $\langle S_{i}\rangle$. The procedure is described in the Appendix A.

Next, we focus on the XMCD data taken at normal incidence (θ=0°), for which the magnetic field is applied along the OZ-axis, B=(0,0,B). For the (checkerboard) antiferromagnetic phase, we use two order parameters $S_{a}$ and $S_{b}$, which represent the OZ-components of the spins in the unit cell as shown in Figure 5a, and minimize the upper bound to the free energy [$F_{\mathrm{AF}}\left(S_{a},S_{b}\right)$] with respect to the order parameters $S_{a}$ and $S_{b}$. Alternatively, one can require stationarity of free energy, $\partial F_{\mathrm{AF}}/\partial S_{a}=0$ and $\partial F_{\mathrm{AF}}/\partial S_{b}=0$, which yields two coupled equations,

(4) $$S_{a}=\frac{\partial F_{1}\left(h_{a}\right)}{\partial h_{a}}\;\;\mathrm{and}\;\;S_{b}=\frac{\partial F_{1}\left(h_{b}\right)}{\partial h_{b}},$$

where $F_{1}$ is the free energy of a single isolated spin. The mean-field solution is obtained from these self-consistent equations. As a rule, several solutions are found. The choice of the physical solution relies again on the lowest value of the free energy. For the superantiferromagnetic phase, we use again two order parameters, $S_{a}$ and $S_{b}$, but now they are distributed in the unit cell as shown in Figure 5b. The mean-field approximation takes into account only the connections (i.e., bonds) between the spins on a local scale, whereas the constrains related to the dimensionality of the systems go unaccounted for. We can, therefore, adapt here all the results derived for the phase in Figure 5a by simultaneously replacing $J_{x}$ and $J_{d}$ in all expressions as

(5) $$\left\{\begin{array}{c} J_{x}\to 2J_{d},\\ J_{d}\to J_{x}/2. \end{array}\right.$$

The factors 2 and 1/2 appear here because each $J_{x}$ connector counts as half a bond in the unit cell, whereas each $J_{d}$ connector counts as a full bond.

We fit the experimental data for normal magnetic fields in Figure 3 assuming the relation $J_{x}=J_{y}=2J_{d}$, which corresponds to the case when a single orbital of the ligand is dominating the superexchange. We reach a good fit to the experimental data for $J_{x}=-0.02$ meV. Our working assumption was that the critical temperature ($T_{N}^{\mathrm{Weiss}}$) is sufficiently low as to allow application of the Weiss theory, i.e., $T_{N}^{\mathrm{Weiss}}<T$. This means also that the order parameters $S_{a}$ and $S_{b}$ are never of opposite sign and are, in fact, equal to each other over the full range of applied magnetic fields. Therefore, the experimental data can equally well be fitted by a ferromagnetic mean-field theory with antiferromagnetic coupling constants. To simplify the matter even further, we consider a square lattice with a single coupling constant *J*. Effectively, this coupling constant will be related to the previous coupling constants by equating to each other $J_{q}$ at q=0 for both models, which immediately yields $4J=2J_{x}+2J_{y}+4J_{d}$. Using the above value, we arrive at $J=3J_{x}/2=-0.03\;\mathrm{meV}$ and D=0.06meV for S=5/2.

The same effective model derived from a mean field Hamiltonian with *J*, *S* and *D* parameters can be used for Ni–TCNQ, although its relation with the microscopic Hamiltonian described in [11] is different. In this case, we find a good fit with J=0.13meV and D=0 for S=1/2.

### 3.3. Spin-Polarized DFT+U Calculations

**Figure 6:**
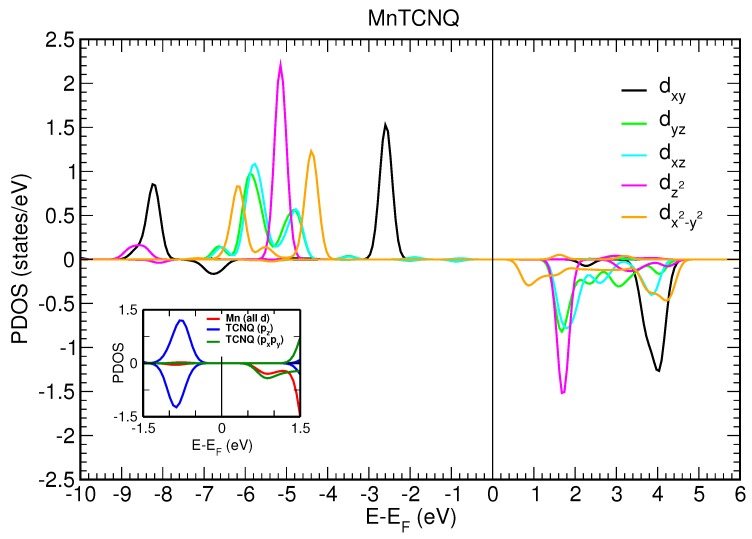
Projected density of states (PDOS) onto the five different Mn(3d) orbitals for Mn–TCNQ. The inset shows the PDOS onto p orbitals of C and N atoms in TCNQ, as well as onto all Mn(3d) orbitals, in a narrow energy range close to the Fermi level ($E_{F}$). Note that the p${}_{z}$ contributions of C and N atoms account for the lowest unoccupied molecular orbital (LUMO).

**Figure 7:**
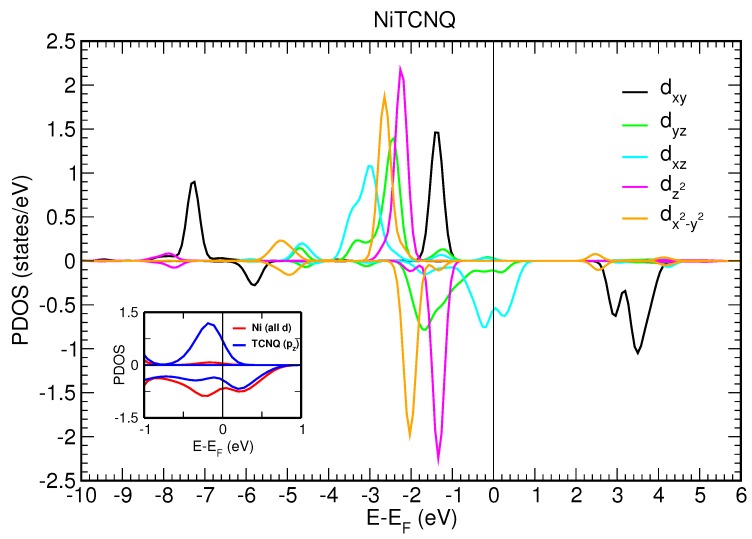
Projected density of states onto the five different Ni(3d) orbitals for Ni–TCNQ. The inset shows the PDOS onto p orbitals of C and N atoms in TCNQ, as well as onto all Ni(3d) orbitals, in a narrow energy range close to the Fermi level ($E_{F}$). Note that the p${}_{z}$ contributions of C and N atoms account for the LUMO.

We first consider a two-dimensional free-standing overlayer description for Mn–TCNQ and Ni–TCNQ networks. Both the lattice vectors and atomic positions have been optimized by using an energy minimization procedure within DFT, as described in the Materials and Methods section. The projected densities of states (PDOS) onto different atomic 3d orbitals of the Mn and Ni atoms are shown in Figure 6 and Figure 7, respectively. The insets show the PDOS onto atomic p orbitals of the C and N atoms of the organic ligand, as well as onto Mn and Ni 3d states without m number resolution, in a narrow energy range close to the Fermi level. A close inspection of Figure 6 and Figure 7 reveals important differences between the two systems under study. The most significant is the half-filling of the 3d states with all the majority spin states occupied in Mn–TNCQ, which corresponds to a value of the spin localized at the Mn atoms approximately equal to S=5/2 . Meanwhile, in Ni–TCNQ only one minority spin state is fully unoccupied (3d${}_{xy}$), which corresponds to a value of the spin localized at the Ni atom of approximately S=1/2, although it can be somewhat higher as the minority spin states 3d${}_{xz}$ and 3d${}_{yz}$ are partially occupied. Additionally, in Ni–TCNQ, the 3d${}_{xz}$ and 3d${}_{yz}$ states are hybridized with TCNQ orbitals close to the Fermi level, in particular the LUMO, giving rise to a delocalized spin density [11]. This can be seen by comparing the PDOS onto atomic p orbitals of the C and N atoms of the TCNQ organic ligand shown in the insets of Figure 6 and Figure 7 for Mn–TCNQ and Ni–TCNQ, respectively. In Ni–TCNQ the LUMO orbital is spin-polarized but this is not the case in Mn–TCNQ, for which the TCNQ LUMO practically does not hybridize with Mn states and is fully occupied. There is another important difference between Ni–TCNQ and Mn–TCNQ: the former is metallic while the second is not. Indeed, the calculated band gap in Mn–TCNQ is rather large (several eV) and translates into large energy barriers for the injection of holes or electrons. As a consequence, electronic charge transfer from the Au(111) surface is expected to play a role in Ni–TCNQ but not in Mn–TCNQ.

**Figure 8:**
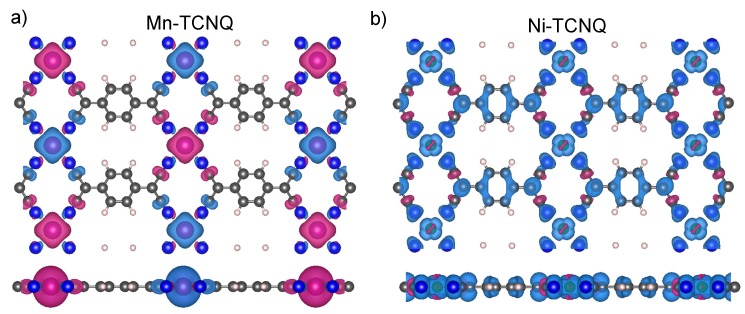
Top (upper panels) and side (lower panels) views of the calculated spin densities for (**a**) Mn–TCNQ and (**b**) Ni–TCNQ free-standing overlayers.

Next, using these two optimized structures calculated with a 1×1 surface unit cell within the DFT+U method with spin polarization as a starting point, we proceed to double the size of the surface unit cell into a 2×1 cell that contains two metal centers (Mn or Ni atoms) and two TCNQ molecules. In this way, we can decide which is the most favorable type of magnetic coupling (ferro- or antiferro-magnetic) between spins localized at the Mn or Ni centers by comparing the values of the corresponding total energies. We consider a checkerboard configuration using oblique vectors in the 2×1 surface unit cell and confirm that ferromagnetic coupling is favorable in Ni–TCNQ networks, while in Mn–TCNQ networks antiferromagnetic coupling is preferred in agreement with [11]. The corresponding spin densities are shown in Figure 8 for Mn–TCNQ and Ni–TCNQ. In Section III of the Supplementary Material we also include other configurations obtained by using a rectangular 2×2 surface unit cell, in which other AFM configurations with spins aligned in rows or columns are considered as well [32], showing the importance of next to nearest neighbors (diagonal) couplings in the networks that have been discussed in the previous section. We have obtained values of *J* using the total energy differences between these frozen spin configurations (see Supplementary Material Section III). The so-calculated values differ with respect to the fitted ones by a factor of five in the case of Mn–TCNQ and by two orders of magnitude in the case of Ni–TCNQ. The large discrepancy found in this latter case of Ni–TCNQ points again towards a more complex scenario than in the Mn–TCNQ case.

It is worth mentioning that the exchange constant *J* obtained in [11] refers to the coupling between the Ni spin and the itinerant spin density of the TCNQ LUMO hybridized band. That exchange coupling is of the direct exchange type and has, therefore, much larger typical values than the mediated couplings (RKKY and superexchange). To emphasize its direct exchange origin we denote it here by $J_{dir}$. A rough estimate for the relation between the two exchange coupling constants can be obtained in terms of the band width of the LUMO hybrid band (*W*) as $J=J_{dir}^{2}/W$. Taking W ∼ 100 meV and the value $J_{dir}=5.55$ meV of [11], we get J∼ 0.3 meV, which has the same order of magnitude as the fitted value.

### 3.4. Magnetocrystalline Anisotropy

The magnetocrystalline anisotropy energies (MAEs) can be obtained from DFT calculations that include SOC effects. The resulting total energies, thus, depend on the orientation of the magnetization density. For extended systems, where the transition metal atomic orbital momentum is expected to be partially or totally quenched, the MAE appears as a second-order SOC effect. In systems where the PDOS is characterized by sharp peaks and devoid of degeneracies at the Fermi level, a second-order perturbative treatment of the SOC makes it possible to establish a few guidelines for the likelihood of an easy axis or plane. The perturbation couples states above and below the Fermi level and it is inversely proportional to the energy difference between states. When the spin-up d-band is completely filled, it can be shown that the energy correction is proportional to the expected value of the orbital magnetic moment and that the spin–flip excitations are negligible [33,34,35].

The total energy variation as a function of the magnetization axis direction is very subtle, often in the sub-meV range per atom. When spin–orbit effects are not strong, it is common practice to use the so-called second variational method [36], where SOC is not treated self-consistently. First, a charge density is converged in a collinear spin-polarized calculation. Next, a new Hamiltonian that includes a SOC term is constructed and diagonalized for two different magnetization directions. Then, the MAE is calculated from the difference of the two band energies. Alternatively, a more precise MAE can be obtained from total energy calculations that include SOC self-consistently. Using the latter method, in this work we have calculated MAE values for free-standing Mn–TCNQ and Ni–TCNQ networks.

The small energies involved in the anisotropy are a challenge for DFT calculations. The MAE is highly sensitive to the geometry and electronic structure calculation details, such as the exchange and correlation functionals and basis set types. From a technical perspective, a reliable MAE is only achieved with demanding convergence criteria. For example, it has been observed that fine k-point samplings of the Brillouin zone are needed [37,38,39]. An account of the convergence details as well as MAE dependence on the *U* parameter can be found in Section IV of the Supplementary Material.

Table 2 shows the obtained values for U=4 eV in 1×1 cells (i.e., only ferromagnetic ordering is considered in this section). For the Mn–TCNQ rectangular network, we find in-plane magnetization with negligible azimuthal dependence, i.e., easy plane anisotropy. The MAE, calculated as the total energy difference between magnetic configurations with Mn magnetizations parallel to the OX and OZ axes, is 0.2 meV. In the Ni–TCNQ rectangular network the energetically preferred magnetization is out-of-plane and the MAE values vary significantly with the azimuthal direction. As shown in Table 2, the values change as much as 1.50 meV with azimuthal angle variations.

The different behavior of the MAE with the azimuthal angle in Mn and Ni networks can be understood in terms of the differences in the metal–molecule bonds, particularly the Mn–N and Ni–N bonds. In both networks the d${}_{x^{2}-y^{2}}$ (with magnetic quantum number m=2), d${}_{xy}\left(m=-2\right)$, and d${}_{z^{2}}\left(m=0\right)$ orbitals remain rather localized, whereas the d${}_{xz}\left(m=1\right)$ and d${}_{yz}\left(m=-1\right)$ orbitals are spread over a wider energy range of a few eV below the Fermi level (see Figure 6 and Figure 7). The delocalization of electronic charge in these d${}_{xz}$ and d${}_{yz}$ orbitals is stronger in the Ni–TCNQ case, where the latter two sub-bands are partially occupied and form hybrid states at the Fermi level with the TCNQ LUMO. As these hybrid states lie at the Fermi level, they have a dominant role in the magnetic anisotropy and, since they yield markedly directional charge and spin density distributions along the Ni–N bonds, they are likely to produce azimuthal MAE variations. Conversely, the Mn d-electrons hybridize weakly with the TCNQ orbitals close to the Fermi level, i.e., with the LUMO, and have essentially no weight at the Fermi level. The spatial extent of these relevant Ni–TCNQ hybrid states is manifested in the delocalized electron spin densities depicted in Figure 8b, as compared to the case of Mn–TCNQ shown in Figure 8a with a spin density more localized at the Mn sites and its neighboring cyano groups.

The existence of an easy axis (plane) of magnetization for Ni (Mn) cannot be anticipated from the electronic structure details. In the Mn–TCNQ system, since the d-band is half filled, the MAE is led by spin–flip excitations and, therefore, the value of the exchange splitting is determinant. In the absence of same-spin excitations, the anisotropy would be associated to the anisotropic part of the spin distribution. More precisely, the MAE would be proportional to the anisotropy of the expected values of the magnetic dipole operator [34,35]. However, Figure 8a shows an anisotropic spin distribution extended towards the cyano groups of the organic ligand TCNQ in the network plane by the crystal field. The quadrupolar moment of this distribution should promote out-of-plane magnetization. This interpretation is at variance with the SOC-self-consistent DFT result. A more elaborated model has been proposed for systems with localized d-orbitals. It states that the spin–flip excitations that keep the quantum number |m| constant favor an in-plane magnetization [40]. The calculated PDOS of Figure 6 shows that the two |m|=2 peaks (d${}_{xy,↑}$ − d${}_{x^{2}-y^{2},↓}$) are those closer to the Fermi level, for majority and minority spin states, respectively. This situation is, in principle, compatible with an easy plane behavior. The conclusion we draw is that the basic qualitative feature of the magnetic anisotropy, namely the magnetization direction, cannot be accounted for by rules of general character, not even in a case like Mn–TCNQ, where the d-electrons have a rather localized character that would make this system seem a priori a good playground for these models.

Next, we turn our attention back to the case of Ni–TCNQ, where the DFT calculations yield a relatively large value for the MAE with out-of-plane magnetization, as well as significant variations of the MAE in the network plane. This theoretical result contrasts with the experimental absence of anisotropy in this system and, thus, requires a further analysis oriented at finding an explanation. As we discuss below, the discrepancy could be explained by substrate effects, mostly due to electronic charge transfer from the metal Au(111) surface. However, if we tried to calculate MAE values from DFT calculations with SOC using the supported Ni–TCNQ/Au(111) model structures presented above, we would not obtain informative results, since it would be very difficult in practice to disentangle the anisotropy effects originated by different aspects of the system. The most significant of them is the unavoidable artificial strain introduced in the system by forcing a commensurable Ni–TCNQ overlayer on top of the Au(111) surface due to the use of periodic boundary conditions in a finite size system imposed by our DFT calculations. However, these limitations can be more conveniently understood using free-standing models.

In the 1×1 rectangular Ni–TCNQ free-standing overlayer we can attribute the large MAE values to the partially occupied Ni(d${}_{xz}$,d${}_{yz}$) states. If these |m|=1 bands were completely filled by transfer of 0.5 electrons from the metallic substrate, their contribution to the MAE would be dramatically reduced. Additionally, the Ni atom spin would become close to S=1/2, a case for which no single-ion anisotropy is possible [29]. However, it is hard to give a precise estimate of the amount of charge transfer and, on top of this, other sources of anisotropy reduction could be at play, like a reduction of Ni coordination due to a geometrical distortion. Indeed, the lowest-energy configuration of this rectangular unit cell is obtained upon a small symmetry-lowering distortion where the four Ni–N bonds are inequivalent: the bonds at $45^{\circ }$ degrees with the OX-axis (d${}_{2}$) have a length of 1.95 Å and the other pair at $-45^{\circ }$ (d${}_{1}$) of 2.01 Å (see Figure 2). The former direction is that of the hardest magnetization axis. This symmetry breaking, though subtle from the geometry point of view, is nevertheless associated to a noticeable asymmetry in the electronic structure, which is in turn behind the strong azimuthal variability of the MAE. In a closer inspection of the PDOS we find that the Ni(d${}_{xz}$,d${}_{yz}$) peaks at the Fermi level hybridized with the molecule LUMO are contributed by d-orbitals lying on the plane containing the short Ni–N bonds (d${}_{2}$) and the surface normal (see Section IV of the Supplementary Material). The long bonds (d${}_{1}$), to which |m|=1 states at the Fermi level do not contribute, correspond to a softer magnetization direction.

To understand the consequences of this distorted geometry on the magnetic anisotropy, we have constructed a free-standing flat Ni–TCNQ model in an oblique unit cell, in which the angle γ between the lattice vectors $\vec{a_{1}}$ and $\vec{a_{2}}$ is varied (the rectangular cell corresponds to $\gamma =90^{\circ }$). As described in Section IV of the Supplementary Material, two cases have been considered: a weakly distorted case with $\gamma =83.5^{\circ }$ and a larger distortion with $\gamma =77.43^{\circ }$. The unit cell angle γ has been reduced while uniformly scaling the lattice constants to keep the unit cell area equal to that of the rectangular equilibrium unit cell. Then, the atomic (x,y) coordinates have been relaxed to satisfy the same convergence criteria as in other models of the present work. For a larger distortion of the rectangular cell with $\gamma =77.43^{\circ }$, one could force a commensurate supercell [(5,2),(1,3)] on Au(111) [4]. In the optimized structure the TCNQ is barely deformed, but one Ni–N bond at the azimuthal direction $\phi =22.6^{\circ }$ is broken because of the cell distortion and the pair of bonds at the $\phi =-75.4^{\circ }$ direction have their lengths reduced to 1.85 Å (see Section IV of the Supplementary Material). The magnetic anisotropy is significantly reduced with respect to that of the rectangular cell, but the hardest direction is still the one along the shortest pair or Ni–N bonds (see Table 2). The main consequence of the Ni coordination reduction caused by the cell shape change is to partially quench its spin. We observe that the local magnetic moment is reduced by about 0.3 $\mu_{B}$, approaching the ideal S=1/2 state that would yield no anisotropy in the single-atom picture. We observe, nevertheless, that this distorted configuration still has partially filled Ni d${}_{xz,yz}\left(|m|=1\right)$ states at the Fermi level (see Section IV of the Supplementary Material). Therefore, we note that this mechanism of anisotropy reduction and the charge transfer effect proposed above are of a different nature, although both originate from the interaction with the substrate.

All in all, the observed lack of magnetic anisotropy in the Ni-TCNQ/Au(111) XMCD data is clearly a substrate effect, which reduces the Ni–TCNQ anisotropy by a combined effect of charge transfer and change of coordination. Nonetheless, other subtle substrate effects not considered here might also have a role, such as fluctuations in the Au–Ni charge transfer due to the incommensurability and corrugation of the network.

## 4. Discussion and Conclusions

Motivated by the XMCD data, we have performed a thorough analysis of the magnetic properties that characterized Mn and Ni metal–organic coordination networks, focusing on the magnetic coupling and anisotropy. By fitting the XCMD data using a model Hamiltonian based on mean-field Weiss theory and comparing with Brillouin functions, we find a completely different behavior for Mn and Ni networks: while in Mn networks the spins localized at the Mn centers are coupled antiferromagnetically with a mild preference to in-plane magnetization, in Ni networks the spins localized at the Ni atoms are coupled ferro-magnetically and do not show any sizable magnetic anisotropy.

These observations are also rationalized with the help of density functional theory calculations in two steps: first we focus on the magnetic coupling and next we address the subtle question of the magnetic anisotropy. Spin-polarized DFT calculations using a 1×1 surface unit cell to describe the free-standing-overlayers reveal a very different electronic structure close to the Fermi level for the two systems under study. The Mn–TCNQ system is insulating and has weak hybridization between Mn and TCNQ states close to the Fermi level, while in Ni–TCNQ, hybridization between Ni (3d) states and the TCNQ LUMO at the Fermi level is rather significant. This difference permits us to explain the observed trends in XMCD data with antiferromagnetic (ferromagnetic) coupling for Mn (Ni) networks that is also confirmed by another set of DFT calculations using a 2×1 surface unit cell.

We find that the basic qualitative feature of the magnetic anisotropy, namely the magnetization direction, cannot be accounted for by rules of general character. Actually, the magneto-crystalline anisotropy is contributed by many electron excitation channels and it clearly shows an intricate dependence on the fine electronic structure details of each particular system. While in Mn–TCNQ/Au(111) the observed magnetic anisotropy with in-plane magnetization agrees with the DFT calculations for the neutral Mn–TCNQ overlayer, the observed lack of magnetic anisotropy in Ni–TCNQ/Au(111) suggests the existence of a substrate effect, which reduces the Ni–TCNQ anisotropy due to a combination of electronic charge transfer and change of Ni–N coordination.

## Figures and Tables

**Table 1:** Moduli of lattice vectors (*a${}_{1}$* and *a${}_{2}$*), angle between lattice vectors (γ), and bond lengths (d${}_{1}$ and d${}_{2}$) of the optimized Mn–TCNQ and Ni–TCNQ 1×1 rectangular and distorted unit cells.

1×1 Cell	Mn–TCNQ	Ni–TCNQ	Ni–TCNQ Oblique 1	Ni–TCNQ Oblique 2
*a${}_{1}$* (Å)	11.52	1.32	11.36	11.46
*a${}_{2}$* (Å)	7.38	7.16	7.18	7.24
$\gamma \;({}^{\circ })$	90	90	83.50	77.43
d${}_{1}$ (Å)	2.12	2.01	1.90	1.84
d${}_{2}$ (Å)	2.12	1.95	2.12	2.00

**Table 2:** Magnetocrystalline anisotropy energies (MAEs, in meV) for Mn– and Ni–TCNQ calculated as the difference $\mathrm{MAE}=E_{tot}\left(0,0\right)-E_{tot}\left(90^{\circ },\phi \right)$, where the two values in parenthesis are the polar and azimuthal angles, respectively, defining the magnetization direction. Positive (negative) energies indicate in-plane (out-of-plane) anisotropy. The last line corresponds to the oblique cell Ni–TCNQ model with angle $\gamma =77.43^{\circ }$, where the anisotropies at the directions of the long (short) pair of Ni–N bond directions are shown. The table values have been obtained for U=4 eV with an energy cutoff of 400 eV and a 12×18×1 k-point sampling, using the tetrahedron method for integration.

	ϕ=0	ϕ=90°	ϕ=45°	ϕ=−45°
Mn	0.20	0.19	0.20	0.20
Ni	−1.44	−0.95	−1.95	−0.45
	ϕ=0	ϕ=90°	ϕ=22.6°	ϕ=−57.4°
Ni (oblique)	−0.07	−0.04	0.03	−0.09

## Supplementary Materials

The following are available online: I. STM data for Mn-TCNQ; II. Supplementary XAS and XMCD data for TCNQ and F4TCNQ networks; III. Energetics of different magnetic configurations using a 2 × 2 unit cell; and IV. MAE convergence details: dependence with U and with lattice distortions.

## Appendix A. Minimization Procedure to Obtain the Self-Consistent Mean Field Equations

The general procedure for the minimization of the free energy of the metal–TCNQ model outlined in Section 3.2 is described here. Given an interacting Hamiltonian *H* and a variational Hamiltonian $H_{0}$, the Bogoliubov upper bound for the free energy reads

(A1) $$\begin{array}{ccc} F & \leq & F_{0}+{\langle H-H_{0}\rangle }_{0}, \end{array}$$

where, by definition,

(A2) $$\begin{array}{c} F=-T\ln Z\;\;\mathrm{and}\;\;F_{0}=-T\ln Z_{0},\\ Z=\mathrm{Tr}\left\{e^{-\beta H}\right\}\;\mathrm{and}\;Z_{0}=\mathrm{Tr}\left\{e^{-\beta H_{0}}\right\},\\ {\langle A\rangle }_{0}=\frac{1}{Z_{0}}\mathrm{Tr}\left\{Ae^{-\beta H_{0}}\right\},\;\;\forall A. \end{array}$$

Using the mean-field Hamiltonian of Equation (3) in the place of $H_{0}$, we obtain the upper bound for the free energy, which needs subsequently to be minimized with respect to the order parameters $\langle S_{i}\rangle$.

To analyze the XMCD data taken at normal incidence, we consider a magnetic field applied along the OZ-axis, B=(0,0,B). The paramagnetic partition function of a single isolated spin reads

(A3) $$Z_{1}\left(h_{z}\right)=\sum_{S_{z}=-S}^{S}e^{-\beta \left(h_{z}S_{z}+DS_{z}^{2}\right)}.$$

The corresponding spin average value can be found in this case by differentiating the free energy ${\langle S\rangle }_{0}=\partial F_{1}/\partial h$, where $F_{1}=-T\ln Z_{1}$.

Two order parameters ($S_{a}$ and $S_{b}$) are needed to describe the (checkerboard) antiferromagnetic phase. They represent the *z*-components of the spins depicted in Figure 5a. The upper bound to the free energy reads (per unit cell):

(A4) $$\begin{array}{ccc} F_{\mathrm{AF}} & = & \left(J_{x}+J_{y}\right)S_{a}S_{b}+J_{d}\left(S_{a}^{2}+S_{b}^{2}\right)+\frac{1}{2}F_{1}\left(h_{a}\right)+\frac{1}{2}F_{1}\left(h_{b}\right)\\ & & -\left(J_{x}+J_{y}\right)\left(S_{a}-\frac{\partial F_{1}\left(h_{a}\right)}{\partial h_{a}}\right)\left(S_{b}-\frac{\partial F_{1}\left(h_{b}\right)}{\partial h_{b}}\right)\\ & & -J_{d}{\left(S_{a}-\frac{\partial F_{1}\left(h_{a}\right)}{\partial h_{a}}\right)}^{2}-J_{d}{\left(S_{b}-\frac{\partial F_{1}\left(h_{b}\right)}{\partial h_{b}}\right)}^{2}, \end{array}$$

with

(A5) $$\begin{array}{ccc} h_{a} & = & g\mu_{B}B-2\left(J_{x}+J_{y}\right)S_{b}-4J_{d}S_{a},\\ h_{b} & = & g\mu_{B}B-2\left(J_{x}+J_{y}\right)S_{a}-4J_{d}S_{b}. \end{array}$$

The terms in the last two lines of Equation (A4) come from the average ${\langle H-H_{0}\rangle }_{0}$ in Equation (A1). These terms are required only when looking for the global minimum of $F_{\mathrm{AF}}\left(S_{a},S_{b}\right)$, which is carried out over the domain $-S\leq S_{a}<S$ and $-S\leq S_{b}<S$. The values of $S_{a}$ and $S_{b}$ at the global minimum then correspond to the mean-field solution. Alternatively, we can use the stationarity condition

(A6) $$\partial F_{\mathrm{AF}}/\partial S_{a}=0\;\;\mathrm{and}\;\;\partial F_{\mathrm{AF}}/\partial S_{b}=0,$$

to obtain the two coupled Equation (4).

These self-consistency equations of the mean-field theory need to be solved for $S_{a}$ and $S_{b}$ by substitution of the expressions for $h_{a}$ and $h_{b}$ from Equation (A5). Since several solutions can be found, we use the condition of least free energy value to select the physical solution. In practice, it is convenient to find a rough approximation for $S_{a}$ and $S_{b}$ by looking for the global minimum of $F_{\mathrm{AF}}\left(S_{a},S_{b}\right)$ on a discrete grid and then refine the obtained solution by iteratively substituting it into the self-consistency Equation (4).
