# Supplementary material for: Magnetic Properties of Metal–Organic Coordination Networks Based on 3d Transition Metal Atoms
Source: Molecules. 2018 Apr 20;23(4):964. doi: 10.3390/molecules23040964 (PMC6017066; doi:10.3390/molecules23040964)
Supplement: Supplementary file 1 [file molecules-23-00964-s001.pdf]

Magnetic properties of metal-organic coordination networks based on 3d transition metal atoms

M. Blanco-Rey,[1,2] A. Sarasola,[3,2] C. Nistor,[4] L. Persichetti,[4] C. Stamm,[4] C. Piamonteze,[5]
P. Gambardella,[4] S. Stepanow,[4] M. M. Otrokov,[6,2,7,8] V. N. Golovach,[1,6,2,9] and A. Arnau[1,6,2]

[1]*Departamento de Física de Materiales UPV/EHU, 20018 Donostia-San Sebastián, Spain*
[2]*Donostia International Physics Center (DIPC), 20018 Donostia-San Sebastián, Spain*
[3]*Departamento Física Aplicada I, Universidad del País Vasco, 20018 Donostia-San Sebastián, Spain*
[4]*Department of Materials, ETH Zürich, Hönggerbergring 64, 8093 Zürich, Switzerland*
[5]*Swiss Light Source, Paul Scherrer Institute, 5232 Villigen PSI, Switzerland*
[6]*Centro de Física de Materiales (CFM-MPC), Centro Mixto CSIC-UPV/EHU, 20018 Donostia-San Sebastián, Spain*
[7]*Tomsk State University, 634050 Tomsk, Russia*
[8]*Saint Petersburg State University, 198504 Saint Petersburg, Russia*
[9]*IKERBASQUE, Basque Foundation for Science, 48013 Bilbao, Basque Country, Spain*

CONTENTS

I. STM image of the Mn-TCNQ network on Au(111) 2

II. Supplementary XAS and XMCD data for the TCNQ and F4TCNQ networks 3

III. Energetics of different magnetic configurations using a 2×2 unit cell 8

IV. MAE convergence details, dependence with U and with lattice distortions 10

References 15

I. STM IMAGE OF THE MN-TCNQ NETWORK ON AU(111)

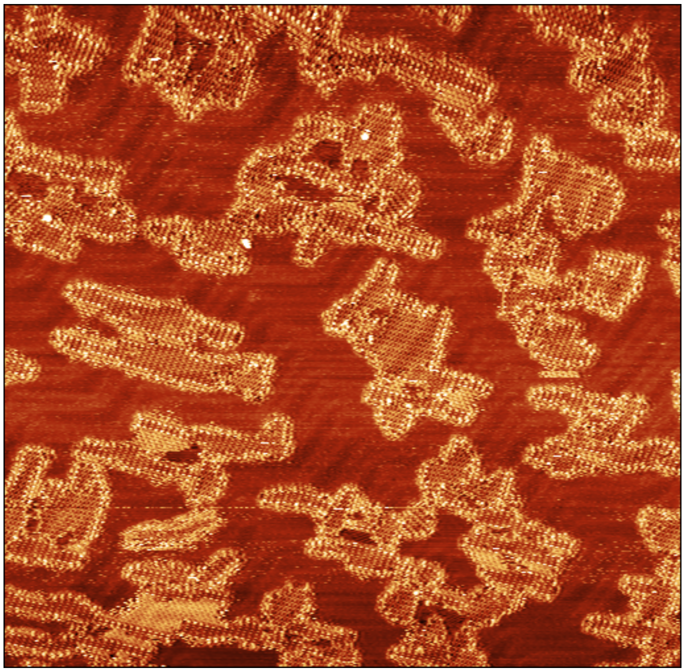

Figure 1. Large scale (120 nm) STM topographical image of the Au(111) surface partially covered by Mn-TCNQ patches. The tunneling conditions were $V = 1.2\ V$ and $I = 0.7\ nA$.

II. SUPPLEMENTARY XAS AND XMCD DATA FOR THE TCNQ AND F4TCNQ NETWORKS

Figures 2 and 3 show the XAS and XMCD data for the Mn-TCNQ, Mn-F4TCNQ, Ni-TCNQ, and Ni-F4TCNQ networks. As F4TCNQ has a stronger electron accepting character than TCNQ, it is employed to manipulate and probe changes in the magnetic coupling between the metal centers. In the case of Mn the XAS and XMCD spectral lineshapes are nearly identical between the two ligand molecule networks, as shown in Figure 4 where the spectra have been directly superposed. In addition, also the magnitude of the XMCD signal does not differ between Mn-TCNQ and Mn-F4TCNQ, indicating no significant influence of the substituted hydrogen atoms by fluorine atoms at the TCNQ backbone on the chemical properties of the Mn centers. Note, that the presented lineshapes are in accordance with the data reported in Ref. [1] , which was acquired at a temperature of 8K, although the magnitude of the XMCD is somewhat larger here as expected when decreasing the temperature. Figure 3 summarizes the XAS and XMCD data recorded for the Ni networks. Again, the XAS and XMCD lineshapes and strengths look very similar for Ni-TCNQ and Ni-F4TCNQ networks. However, for the F4TCNQ ligand one notices a shoulder on the high energy side of the L_3-edge. This can be due to a charge-transfer satellite peak in the XAS, which is shifted to higher energies for the stronger electron acceptor molecule. This difference is less pronounced in the XMCD lineshapes. Note, that also in this case the reported data are in accordance with earlier reported results for measurements at 8K [1, 2], having nearly the same XMCD magnitude here at 2.5 K. For a direct comparison of the Ni-TCNQ and Ni-F4TCNQ XAS and XMCD lineshapes see Figure 5.

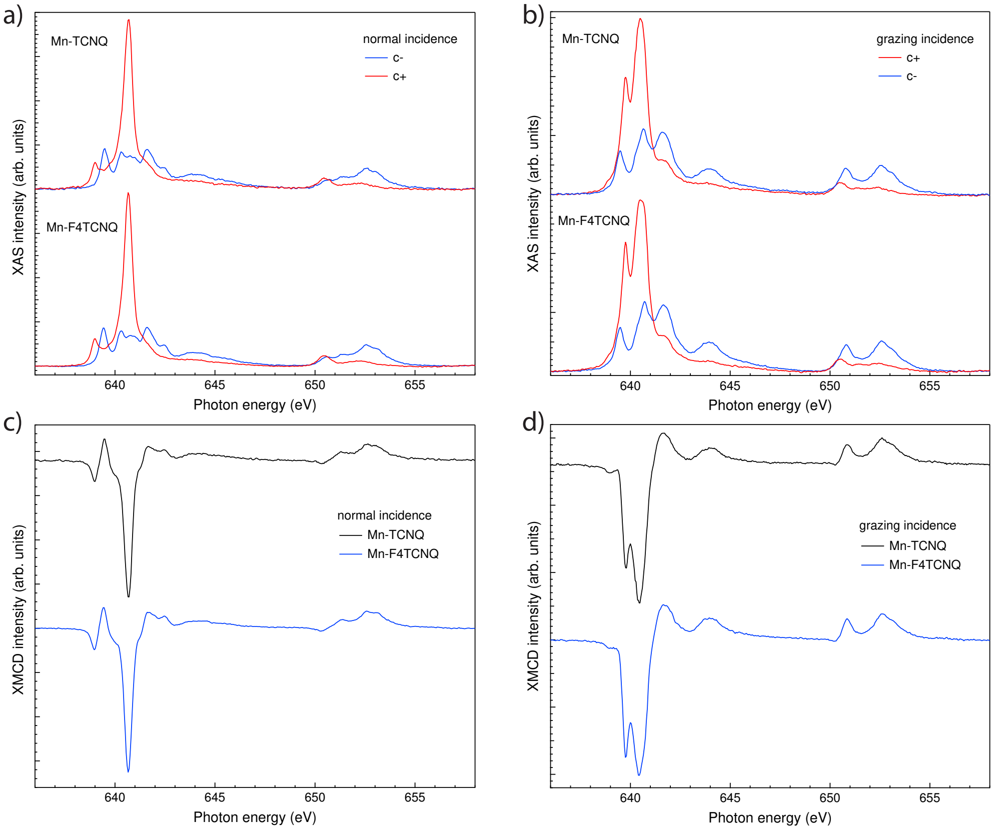

Figure 2. XAS (a and b) with circular polarization and XMCD (c and d) data at normal ($\theta = 0°$) and grazing ($\theta = 60°$) x-ray incidence for the networks Mn-TCNQ and Mn-F4TCNQ. $B = 6.8$ T, $T = 2.5$ K.

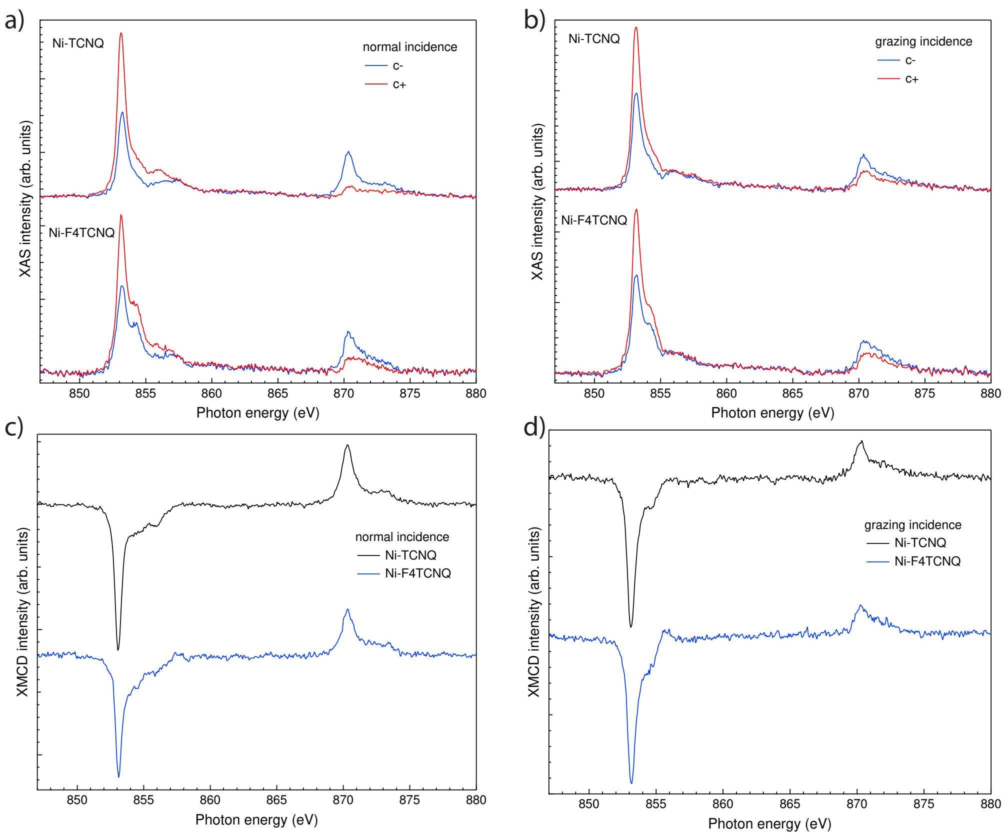

Figure 3. XAS (a and b) with circular polarization and XMCD (c and d) data at normal ($\theta = 0\circ$) and grazing ($\theta = 60°$) x-ray incidence for the networks Ni-TCNQ and Ni-F4TCNQ. $B = 6.8$ T, $T = 2.5$ K.

The corresponding magnetization curves of the Mn and Ni centers are presented in Figure 6 a) and b), respectively. The data exhibits minor changes between the TCNQ and F4TCNQ ligands. Figure 6 a) also shows the magnetization curve for the pure Mn/Au(111) system, which was prepared in the same way as the Mn-networks, but with no molecules. One clearly sees that the magnetization curve for Mn embedded in the organic adlayer is much less S-shaped in accordance with the anti-ferromagnetic coupling. The data further indicates that the AFM coupling is reduced when using the F4TCNQ ligand but the anisotropy remains. In contrast, the Ni magnetization curves (Figure 6 b)) show only minor differences between TCNQ and F4TCNQ with a slightly less steep slope, i.e. a lower susceptibility, for F4TCNQ in the low field region. The high field region is very similar. Also for F4TCNQ we observe an isotropic behavior of the magnetization. Note, that Ni impurities on Au(111) are non-magnetic [2].

As a side result, we further attempted to modify the electronic structure of the metal centers and hence their magnetic coupling by the adsorption of oxygen molecules on the network structures. However, the results reproduced in Figure 7 indicate no or only minor changes in the XAS and XMCD lineshapes and magnitudes, which hints that the metal-organic networks are not susceptible to oxygen atmospheres (partial pressure 8×10^{-8} mbar, dosing time 30 min), i.e., molecular oxygen does not chemisorb under the employed conditions on the metal centers. For comparison, the data for the pure Mn/Au(111) impurity system has been added to Figure 7. The lineshape of the metallic Mn is clearly distinct from the Mn embedded in the metal-organic networks. The absence of oxygen-induced changes to the magnetic properties is further evidenced in the magnetization curves shown in Figure 8, where we find essentially no significant changes.

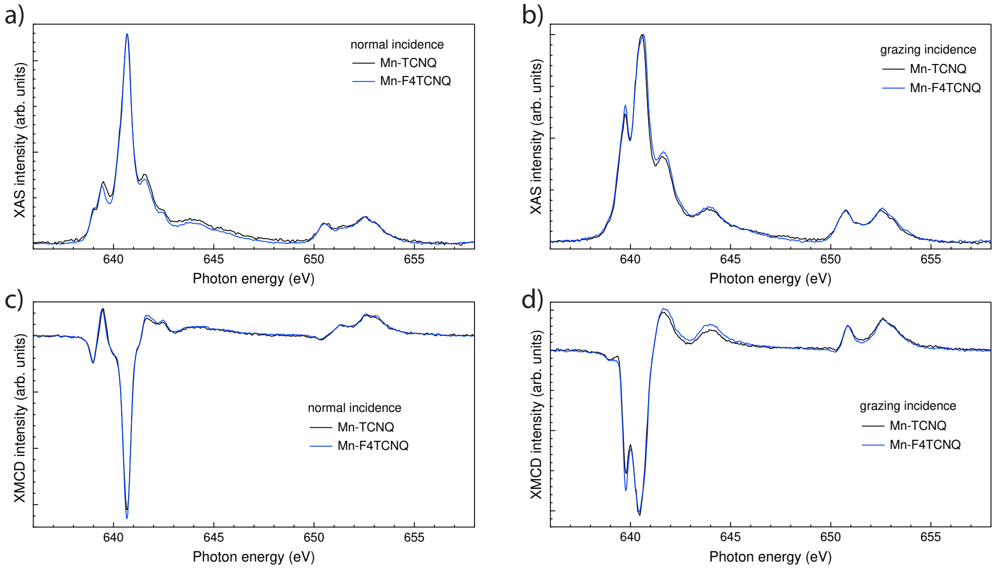

Figure 4. Comparison of the XAS and XMCD lineshapes acquired for the Mn-TCNQ and Mn-F4TCNQ networks.

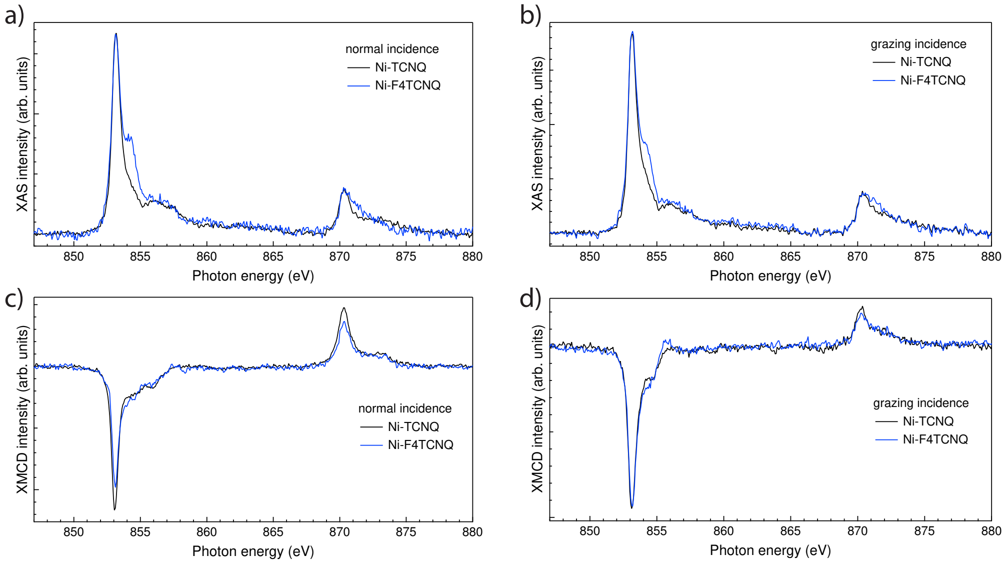

Figure 5. Comparison of the XAS and XMCD lineshapes acquired for the Ni-TCNQ and Ni-F4TCNQ networks.

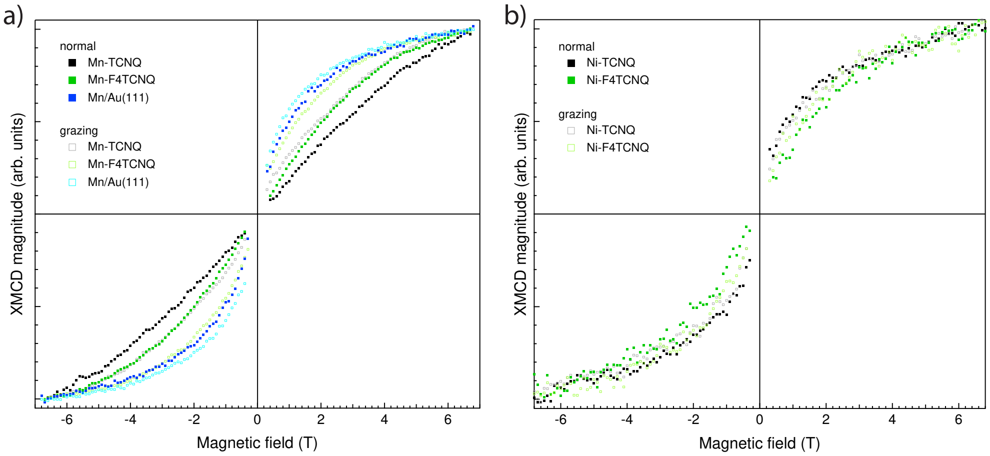

Figure 6. Magnetization curves acquired at normal and in-plane (grazing) magnetic fields for a) Mn-TCNQ and -F4TCNQ networks and pure Mn/Au(111) impurity system as well as b) Ni-TCNQ and -F4TCNQ networks. For comparison of the shapes the magnetization curves have been normalized to the same value at the highest magnetic field.

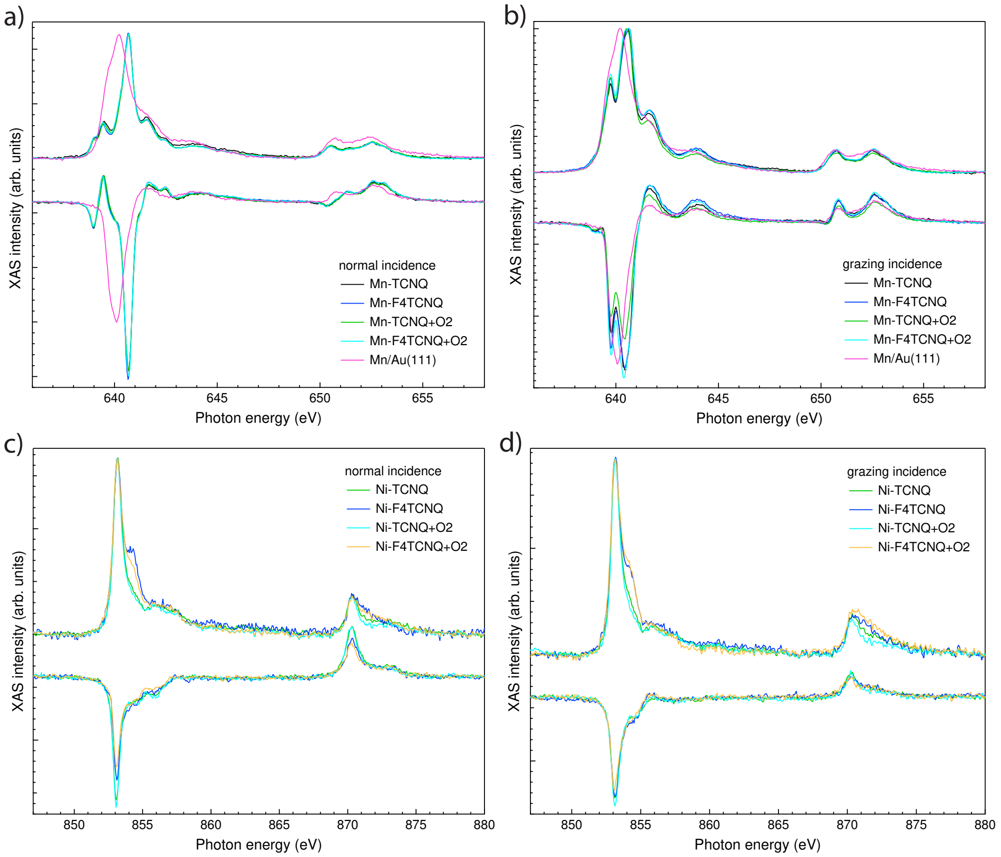

Figure 7. Comparison of the XAS and XMCD spectra for the Mn and Ni networks before and after oxygen exposure. The data of the pure Mn/Au(111) impurity system is also shown.

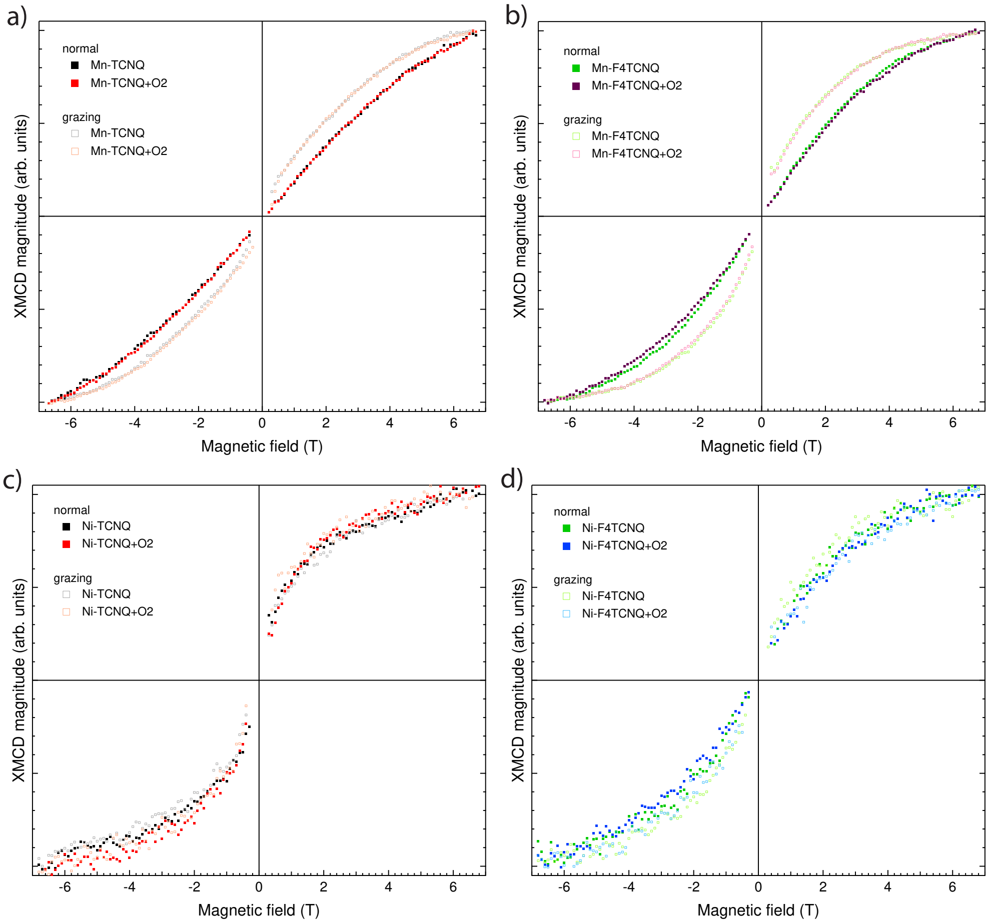

Figure 8. Magnetization curves for normal and in-plane (grazing) magnetic fields for the different metal-organic networks before and after oxygen exposure.

III. ENERGETICS OF DIFFERENT MAGNETIC CONFIGURATIONS USING A 2×2 UNIT CELL

In this section, we complement the results presented in the core of the paper for the more favorable magnetic configurations, i.e., AFM or FM coupling, of Mn-TCNQ and Ni-TCNQ free-standing overlays using a 2×2 supercell that permits to consider three different AFM configurations (described below) and the FM configuration with aligned spin moments. In addition, we also include the results of calculations done for the analogue systems Mn-F4TCNQ and Ni-F4TCNQ. Using spin polarized DFT+U, as described in the Materials and Methods section, we have calculated the energy of the four different configurations shown in Figure 9. In the case of Ni networks, we have considered a charged free-standing overlayers with an additional electron, i.e., 0.25 e^- per Ni atom, to account for the electronic charge transferred from the Au(111) surface.

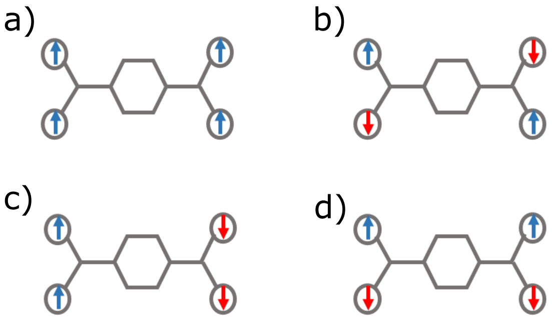

Figure 9. Simplified structure of the different magnetic configurations that have been considered: a) Ferromagnetic coupling. b) Antiferromagnetic checker board coupling. c) Antiferromagnetic coupling in columns. d) Antiferromagnetic coupling in rows.

The magnetic interaction energies corresponding to these configurations are given in terms of the exchange coupling constants defined in Figure 5 of the main paper (J_x, J_y, and J_d) and the value of the localized spin (S) by

$$a)\ E_{FM} = (-J_x - J_y - 2J_d)S^2$$
$$b)\ E_{CB} = (J_x + J_y - 2J_d)S^2$$
$$c)\ E_{col} = (J_x - J_y + 2J_d)S^2$$
$$d)\ E_{row} = (-J_x + J_y + 2J_d)S^2$$

Table I. Total energy differences of the four different magnetic configurations considered.

MOCN	2x2 cell	$E_i - E_{most\ stable}$(eV)	$m_{metal}(\mu_B)$
Mn-TCNQ(neutral)	AFM checker board	0.00002	$\pm$ 4.573
	AFM rows	0.00000	$\pm$ 4.573
	AFM columns	0.01259	$\pm$ 4.575
	FM	0.01391	+ 4.575
Mn-F_4TCNQ(neutral)	AFM checker board	0.00009	$\pm$ 4.531
	AFM rows	0.00000	$\pm$ 4.531
	AFM columns	0.01660	$\pm$ 4.534
	FM	0.01840	+ 4.535
Ni-TCNQ(charged)	AFM checker board	0.06007	$\pm$ 1.279
	AFM rows	0.10523	$\pm$ 1.285
	AFM columns	0.06648	$\pm$ 1.282
	FM	0.00000	+ 1.268
Ni-F_4TCNQ(charged)	AFM checker board	0.03954	$\pm$ 1.360
	AFM rows	0.08801	$\pm$ 1.366
	AFM columns	0.06613	$\pm$ 1.367
	FM	0.00000	+ 1.355

In the case of Mn-TCNQ and Mn-F4TCNQ networks, as shown in Table I , the most favorable magnetic configuration corresponds to AFM coupling between Mn spins, being degenerate the checker-board and the AFM rows configurations. The energy difference with the other magnetic configurations considered is of the order of a few meV / Mn atom. However, in Ni-TCNQ and Ni-F4TCNQ networks, we find a clear energetic preference for the FM configurations by about 10 and 15 meV /Ni atom, respectively. Therefore, we see that these results are consistent with the basic trends observed in the XMCD data: (i) favorable AFM or FM magnetic coupling for Mn or Ni or Mn networks, respectively, (ii) weaker coupling in Mn networks than in Ni networks, and (iii) same qualitative behavior in TCNQ and F4TCNQ networks.

After using one of the equations above for the energy reference value, we arrive at a system of three linear equations, which can be easily solved, yielding J_x, J_y, and J_d in terms of the total energy differences. By substitution of the calculated DFT values of Table I in the equations, we obtain those exchange coupling constants and, finally, the effective value given by $J = (J_x + J_y)/2 + J_d$.

Table II. Exchange couplings obtained from DFT total energies of 2×2 configurations (in meV).

**MOCN**	**S**	J_x	J_y	J_d	J
Mn-TCNQ(neutral)	5/2	-0.013	-0.27	-0.0065	-0.15
Ni-TCNQ(charged)	1/2	5.38	24.63	3.48	13.94

IV. MAE CONVERGENCE DETAILS, DEPENDENCE WITH U AND WITH LATTICE DISTORTIONS

The tables show the magnetic anisotropy energy values with respect to normal direction calculated as MAE = $E_{tot}(0,0) - E_{tot}(90°, \phi)$, where the polar and azimuthal angles, respectively, appear in parenthesis. Positive (negative) MAE values indicate in-plane (out-of-plane) anisotropy.

In some of the Ni-TCNQ cases, the MAE calculations with large basis sets could not be converged self-consistently with the added spin-orbit coupling. For these situations, we constrain the direction of the Ni local magnetization. The energy associated to this constraint is $\sim 0.2 \times 10^{-6}$ eV, only slightly larger than the chosen total energy tolerance (10^{-7} eV). Therefore, these values should be taken with caution as they do not have a comparable accuracy to the others.

Note that the details of the electron density at the Fermi level of Ni-TCNQ are responsible for the strong dependence on the integration method, which is not observed in Mn-TCNQ (compare Tables III and VI). Thus, we use the accurate tetrahedron method with Blöchl corrections to obtain the MAE values.

Table III. Convergence test of MAE values, in meV, for rectangular Mn-TCNQ with lattice constants $a = 11.52, b = 7.38$ Å and $U = 4$ eV. In bold, the values considered as well converged, which are given in the main text. In this table different cut-off energies, k-point meshes, and integration method of the Fermi level are considered. For the latter, the given numbers are the gaussian smearing widths.

$E_{cut-off}$ (eV)	k-points	integration	$\phi = 0$	$\phi = 90°$	$\phi = 45°$	$\phi = -45°$
375	$8 \times 12 \times 1$	tetrahedron	0.20	0.19		
400	$8 \times 12 \times 1$	tetrahedron	0.20	0.19		
400	$8 \times 12 \times 1$	0.10 eV	0.20	0.19		
400	$8 \times 12 \times 1$	0.05 eV	0.20	0.19		
425	$8 \times 12 \times 1$	tetrahedron	0.20	0.19		
**400**	$12 \times 18 \times 1$	**tetrahedron**	**0.20**	**0.19**	**0.20**	**0.20**
400	$12 \times 18 \times 1$	0.10 eV	0.20	0.19	0.20	0.20
400	$12 \times 18 \times 1$	0.05 eV	0.20	0.19	0.20	0.20
400	$18 \times 24 \times 1$	tetrahedron	0.20	0.19		

Table IV. MAE values, in meV, as a function of the U parameter for rectangular Mn-TCNQ. Lattice constants are fixed at $a = 11.52, b = 7.38$ Å and the convergence settings marked in bold in Table III are chosen for the calculations. The values given in bold here correspond to the result in the main paper, obtained for $U = 4$ eV.

U (eV)	$\phi = 0$	$\phi = 90°$
2	0.28	0.27
3	0.23	0.23
**4**	**0.20**	**0.19**
5	0.18	0.17
6	0.15	0.15

Table V. MAE values for Mn-TCNQ, in meV, as a function of lattice expansions or contractions that keep the unit cell rectangular shaped. We take $U = 4\,\text{eV}$ and the convergence settings marked in bold in Table III are chosen for the calculations. The values given in bold here correspond to the result in the main paper, obtained for the equilibrium lattice constants $a = 11.52, b = 7.38\,\text{Å}$.

strain	$\phi = 0$	$\phi = 90°$
-2%	0.30	0.29
**0**	**0.20**	**0.19**
2%	0.14	0.13

Table VI. Convergence test of MAE values, in meV, for rectangular Ni-TCNQ with lattice constants $a = 11.32, b = 7.16\,\text{Å}$ and $U = 4\,\text{eV}$. In bold, the values considered as well converged, which are given in the main text. In this table different cut-off energies, k-point meshes, and integration method of the Fermi level are considered. For the latter, the given numbers are the first-order Methfessel-Paxton smearing widths. The values in parenthesis for $18 \times 24 \times 1$ are calculated with constrained direction of the Ni local magnetization along OX and OY, respectively.

$E_{cut-off}$ (eV)	k-points	integration	$\phi = 0$	$\phi = 90°$	$\phi = 45°$	$\phi = -45°$
375	$8 \times 12 \times 1$	tetrahedron	-1.46	-0.95	-1.96	-0.42
400	$8 \times 12 \times 1$	tetrahedron	-1.42	-0.94	-1.92	-0.41
400	$8 \times 12 \times 1$	0.10 eV	-1.47	-0.79	-1.95	-0.43
400	$8 \times 12 \times 1$	0.05 eV	-1.42	-0.77	-1.91	-0.40
425	$8 \times 12 \times 1$	tetrahedron	-1.46	-0.84	-1.89	-0.39
**400**	$12 \times 18 \times 1$	**tetrahedron**	**-1.44**	**-0.95**	**-1.95**	**-0.45**
400	$12 \times 18 \times 1$	0.10 eV	-1.18	-0.64	-1.93	-0.42
400	$12 \times 18 \times 1$	0.05 eV	-1.10	-0.51	-1.91	-0.42
400	$18 \times 24 \times 1$	tetrahedron	(-1.50)	(-0.98)	-1.97	-0.46

Table VII. MAE values, in meV, as a function of the U parameter for rectangular Ni-TCNQ. Lattice constants are fixed at $a = 11.32, b = 7.16\,\text{Å}$ and the convergence settings marked in bold in Table VI are chosen for the calculations. The values given in bold here correspond to the result in the main paper, obtained for $U = 4\,\text{eV}$.

U (eV)	$\phi = 0$	$\phi = 90°$	$\phi = 45°$	$\phi = -45°$
2	-2.53	-1.37	-2.54	-1.60
3	-2.60	-1.52	-2.68	-1.43
**4**	**-1.44**	**-0.95**	**-1.95**	**-0.45**
5	-0.81	-0.46	-1.39	0.08
6	-0.60	-0.38	-1.18	0.23

Table VIII. MAE values for Ni-TCNQ, in meV, as a function of lattice expansions or contractions that keep the unit cell rectangular shaped. We take $U = 4\,\text{eV}$ and the convergence settings marked in bold in Table VI are chosen for the calculations. The values given in bold here correspond to the result in the main paper, obtained for the equilibrium lattice constants $a = 11.32, b = 7.16\,\text{Å}$.

strain	$\phi = 0$	$\phi = 90°$	$\phi = 45°$	$\phi = -45°$
-2%	-3.26	-2.29	-2.90	-2.58
-1%	-2.01	-1.20	-2.16	-1.13
**0**	**-1.44**	**-0.95**	**-1.95**	**-0.45**
1%	-1.06	-0.47	-1.72	0.10
2%	-0.87	-0.14	-1.66	0.46

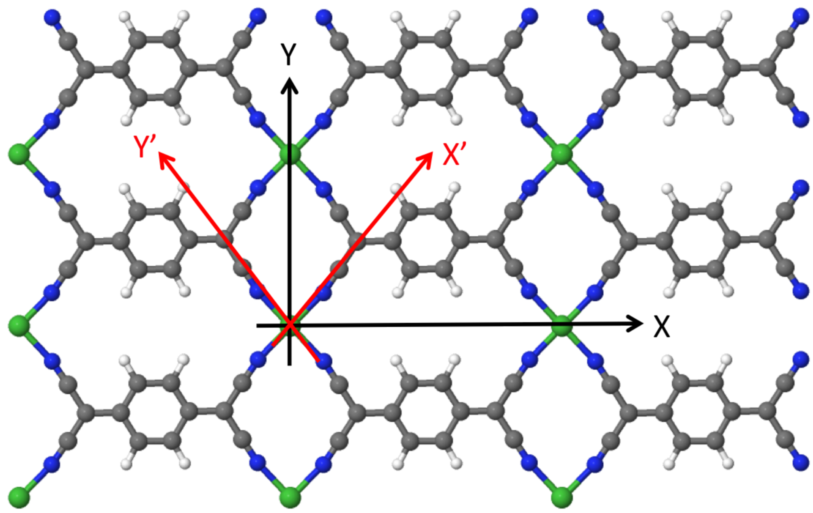

Figure 10. Top view of the Ni-TCNQ network model with rectangular unit cell used in this work. The Ni-N bond length along the OX' (OY') axis is $d_2 = 1.95$ Å ($d_1 = 2.01$ Å).

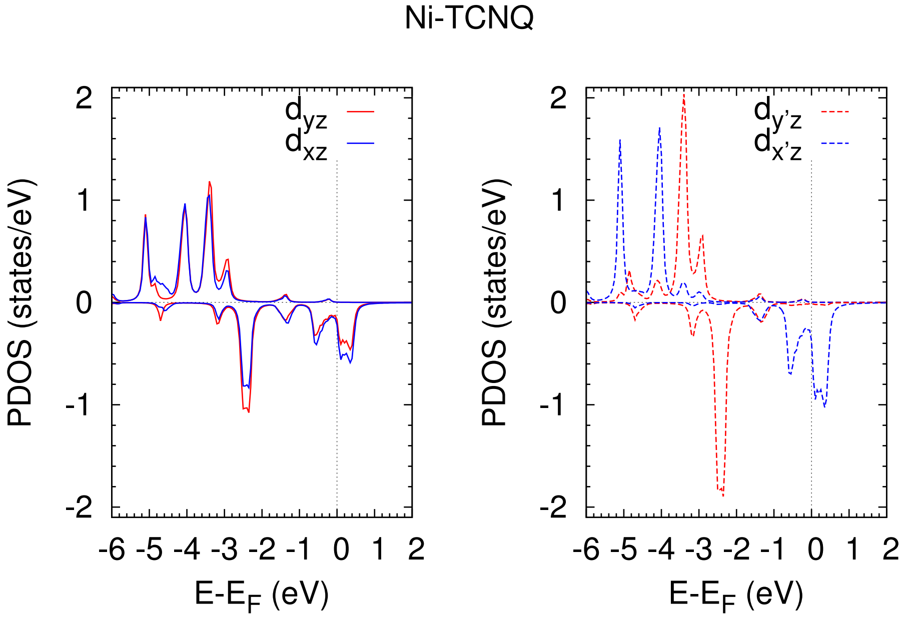

Figure 11. Projected density of states (PDOS) onto the d_{xz} and d_{yz} orbitals of the Ni for the two axes conventions shown in Fig. 10.

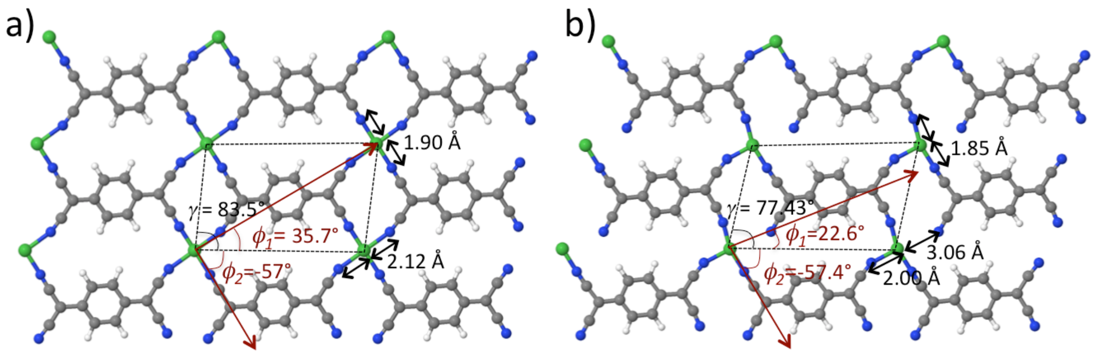

Figure 12. Ni-TCNQ network models with oblique unit cells used in this work.

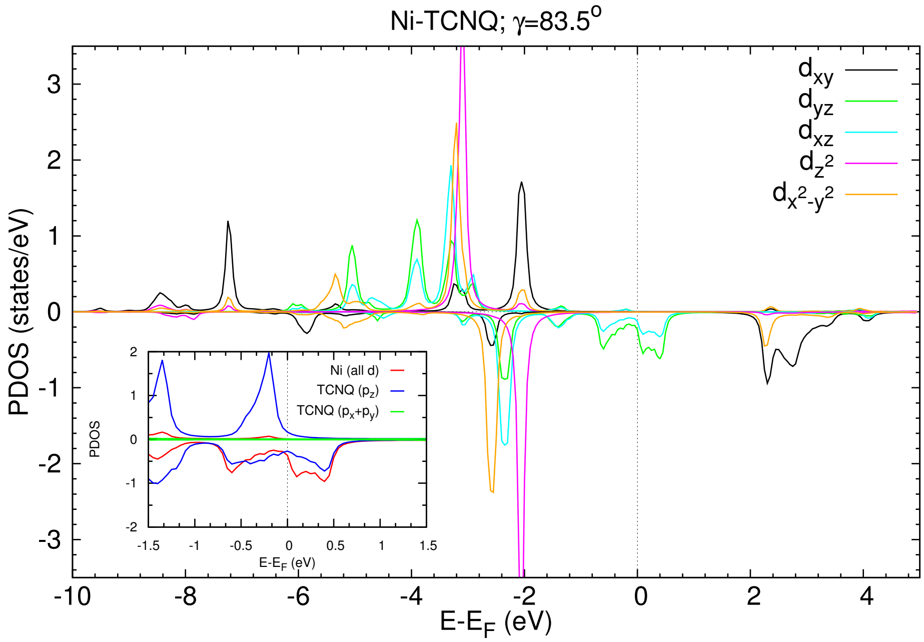

Figure 13. Projected density of states (PDOS) onto the five Ni(3d) orbitals for the oblique cell model with internal angle $\gamma = 83.5°$. The inset shows the PDOS onto the p orbitals of the C and N atoms of the TCNQ molecule.

Table IX. MAE values for Ni-TCNQ, in meV, as a function of a shear distortion of the lattice that keep the unit cell area equal to that of the rectangular equilibrium cell. γ is the unit cell angle. We take $U = 4\,\text{eV}$ and the convergence settings marked in bold in Table VI are chosen for the calculations. The considered azimuthal directions ϕ_i are shown in Figure 12.

γ	$\phi = 0$	$\phi = 90°$	ϕ_1	ϕ_2
83.5°	-0.35	-0.55	0.38	-1.06
77.43°	-0.07	-0.04	0.03	-0.09

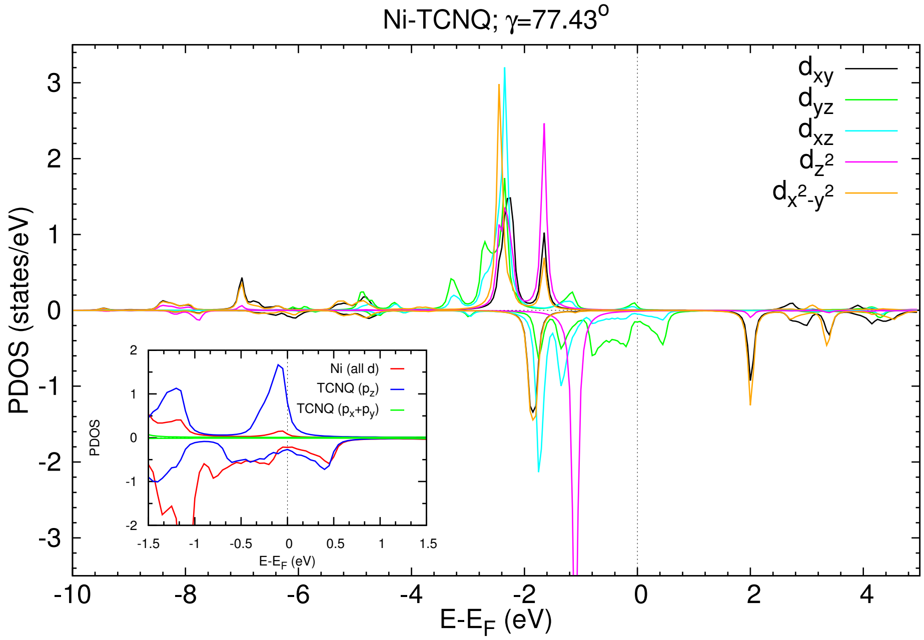

Figure 14. Same as Fig. 13 for the oblique cell with angle $\gamma = 77.43°$.

[1] M. N. Faraggi, V. N. Golovach, S. Stepanow, T.-C. Tseng, N. Abdurakhmanova, C. S. Kley, A. Langner, V. Sessi, K. Kern, and A. Arnau, The Journal of Physical Chemistry C **119**, 547 (2015).
[2] N. Abdurakhmanova, T.-C. Tseng, A. Langner, C. S. Kley, V. Sessi, S. Stepanow, and K. Kern, Phys. Rev. Lett. **110**, 027202 (2013).
